# Methanotroph-methylotroph lipid adaptations to changing environmental conditions

**DOI:** 10.3389/fmicb.2025.1532719

**Published:** 2025-02-07

**Authors:** Nora Richter, Laura Villanueva, Ellen C. Hopmans, Nicole J. Bale, Jaap S. Sinninghe Damsté, Darci Rush

**Affiliations:** ^1^Department of Marine Microbiology and Biogeochemistry, NIOZ Royal Netherlands Institute for Sea Research, Den Burg, Netherlands; ^2^Department of Biology, Faculty of Science, Utrecht University, Utrecht, Netherlands; ^3^Department of Earth Sciences, Faculty of Geosciences, Utrecht University, Utrecht, Netherlands

**Keywords:** bacteriohopanepolyols, methane-oxidizing bacteria, respiratory quinones, membrane lipids, methylotroph

## Abstract

Methanotrophs, in particular methane-oxidizing bacteria (MOB), regulate the release of methane from lakes, and often co-occur with methylotrophs that may enhance methane-oxidation rates. Assessing the interaction and physiological status of these two microbial groups is essential for determining the microbial methane buffering capacity of environmental systems. Microbial membrane lipids are commonly used as taxonomic markers of specific microbial groups; however, few studies have characterized the changes of membrane lipids under different environmental conditions. For the case of methane-cycling microorganisms, this could be useful for determining their physiological status and potential methane buffering capacity. Here we investigated the changes in membrane lipids, bacteriohopanepolyols (BHPs) and respiratory quinones, produced by MOB and methylotrophs in an enrichment co-culture that primarily consists of a methanotroph (*Methylobacter* sp.) and a methylotroph (*Methylotenera* sp.) enriched from a freshwater lake under different methane concentrations, temperatures, and salinities. To assess whether the lipid response is similar in methanotrophs adapted to extreme environmental conditions, we also characterize the BHP composition and respiratory quinones of a psychrotolerant methanotroph, *Methylovulum psychrotolerans*, isolated from an Arctic freshwater lake and grown under different temperatures. Notably, in the *Methylobacter-Methylotenera* enrichment the relative abundance of the BHPs aminobacteriohopanepentol and aminobacteriohopanepolyols with additional modifications to the side chain increased at higher temperatures and salinities, respectively, whereas there was no change in the distribution of respiratory quinones. In contrast, in the *Methylovulum psychrotolerans* culture, the relative abundance of unsaturated BHPs increased and ubiquinone 8:8 (UQ_8:8_) decreased at lower temperatures. The distinct changes in lipid composition between the *Methylobacter-Methylotenera* enrichment and the psychrotolerant methanotroph at different growth temperatures and the ability of the *Methylobacter-Methylotenera* enrichment to grow at high salinities with a singular BHP distribution, suggests that methane-cycling microbes have unique lipid responses that enable them to grow even under high environmental stress.

## 1 Introduction

Methane-oxidizing bacteria (MOB) modulate the natural release of methane (CH_4_), a potent greenhouse gas, through aerobic methane oxidation (see Hanson and Hanson, 1996 for a review). MOB belong to the phylum Proteobacteria in the classes Gammaproteobacteria (Type I and Type X methanotrophs) and Alphaproteobacteria (Type II methanotrophs; Hanson and Hanson, 1996; Bowman, 2006), and were also identified in the phyla Verrucomicrobia (Dunfield et al., 2007; Pol et al., 2007; Islam et al., 2008) and NC10 (Raghoebarsing et al., 2006; Ettwig et al., 2009). Type I members of Gammaproteobacteria are widespread in both terrestrial and marine environments (Knief, 2015). In lakes, for instance, Type I methanotrophs are the primary methane-oxidizers in both the water column and surface sediments (Hanson and Hanson, 1996). Members of the methylotrophic (i.e., microorganisms that consume single carbon compounds) *Methylotenera* genus are known to co-occur with methanotrophs, and under nitrate-rich conditions are thought to play a role in enhancing methane oxidation rates by the removal of toxic products (e.g., methanol and formaldehyde) that inhibit methanotrophy (Mustakhimov et al., 2013; Krause et al., 2017; Yu and Chistoserdova, 2017; van Grinsven et al., 2020). The capacity for methanotrophs to regulate methane emissions from lakes is, thus, linked to microbial interactions with methylotrophs, as well as the physiological ability of both methanotrophs and methylotrophs to cope with environmental stress. In microbes, the physiological response to external stress is regulated by membrane lipids, such as bacteriohopanepolyols (BHPs; see Belin et al., 2018 and Newman et al., 2016 for a review) and respiratory quinones (see Franza and Gaudu, 2022 for a review); therefore, membrane lipids are crucial for understanding the functional potential of methane-cycling microbes.

In gram-negative bacteria, BHPs are found in the inner and outer membrane (e.g., Jahnke et al., 1992; Jürgens et al., 1992; Doughty et al., 2009; Wu et al., 2015), and play an important physiological role in regulating the permeability and rigidity of the cell membrane in response to external environmental stress (Welander et al., 2009; Doughty et al., 2011; Schmerk et al., 2011). BHPs are structurally diverse compounds with unique side-chain modifications that are thought to be specific to certain microbes (Rohmer et al., 1984; Talbot et al., 2003; Talbot and Farrimond, 2007; Kusch and Rush, 2022). For instance, 35-aminobacteriohopane-30,31,32,33,34-pentol (aminopentol from herein) and 35-aminobacteriohopane-31,32,33,34-tetrol (aminotetrol from herein) are considered specific to Type I and Type II methanotrophs, respectively (Neunlist and Rohmer, 1985; Cvejic et al., 2000; Talbot et al., 2001). Incubation experiments with methanotrophs further suggest that concentrations and relative abundances of aminotetrol, aminopentol, and their unsaturated counterparts vary in relation to temperature (Jahnke et al., 1999; Osborne, 2015; Osborne et al., 2017; Bale et al., 2019; van Winden et al., 2020), and, therefore, might be involved in maintaining the fluidity of the cell membrane. Further work is needed, however, to determine whether the BHP response in methanotrophs is the same across different species and various environmental factors.

Respiratory quinones are isoprenoidal-based membrane lipids associated with metabolic processes in eukaryotes, bacteria, and archaea, as they are essential components of electron transport chains involved in electron and proton shuttling within the cytoplasmic membrane (Anraku, 1988). Respiratory quinones are characterized by a polar cyclic headgroup and isoprenoid side chain that imparts a redox potential and can be adapted to certain metabolic processes (Nowicka and Kruk, 2010). As such, respiratory quinones have been used as metabolic markers for redox processes (e.g., Dupont et al., 2014; Becker et al., 2018), quantitative measures of bacterial biomass (Hiraishi et al., 1998; Saitou et al., 1999), and as chemotaxonomic biomarkers (Collins and Green, 1985; Hiraishi, 1999). Further studies suggest that respiratory quinones might also be involved in regulating membrane fluidity under osmotic stress (Sévin and Sauer, 2014; Eriksson et al., 2019), oxidative stress (Søballe and Poole, 1999), and at low temperatures (Seel et al., 2018). So far, the role of respiratory quinones in stress resistance for methane-cycling microbes has not been evaluated.

Continuous advancements in analytical techniques and their application to environmental samples and cultures has led to the discovery of many new membrane lipids (e.g., Talbot et al., 2016; Hopmans et al., 2021). For instance, the analysis of underivatized BHPs using ultra high pressure liquid chromatography (UHPLC) coupled to electrospray ionization (ESI)-high resolution dual-stage mass spectrometry (HRMS^2^) led to the identification of many novel BHPs (Hopmans et al., 2021). Similarly, sample analyses for respiratory quinones using an UHPLC system equipped with ESI revealed a wide array of respiratory quinones in environmental samples (e.g., Becker et al., 2018). The application of these analytical techniques to study membrane lipids, has the potential to uncover novel lipids that could provide new insights into environmental stress resistance.

In this study, we use UHPLC-HRMS^2^ to characterize how BHP and respiratory quinone distributions in an enrichment culture, consisting of a dominant methanotroph (*Methylobacter* sp.) and a methylotroph (*Methylotenera* sp.) that metabolically interact and were obtained from a eutrophic lake (van Grinsven et al., 2020), vary in response to changing environmental conditions. We use an enrichment co-culture to assess how the microbial community responds to external environmental stress and provide context for how this might influence microbial interactions in a lake environment. Further, we evaluate BHPs for their biomarker potential in methanotrophs, and we assess changes in respiratory quinones to understand how the redox status of the cells varies in response to external stress. To determine whether methanotrophs adapted to extreme environments have a similar physiological response to changing temperatures, we also analyzed the lipid composition of a psychrotolerant methanotroph, *Methylovulum psychrotolerans*, isolated from an Arctic freshwater lake (Oshkin et al., 2016; Bale et al., 2019).

## 2 Methods

### 2.1 *Methylobacter-Methylotenera* enrichment culture

The enrichment co-culture was previously isolated from a hypereutrophic, monomictic lake (Lacamas Lake, WA, United States; van Grinsven et al., 2020). Prior to setting up the incubation experiments the enrichment was grown at 15°C in oxic and dark conditions with nitrate mineral salts (NMS) media (Whittenbury et al., 1970). Incubation experiments were set up to observe how methane concentration, temperature, and salinity affect the BHP lipidome of the enrichment co-culture (see Supplementary Table S1 for experimental conditions). All experiments were set up in triplicate in 580 mL acid-washed and autoclaved glass bottles with butyl rubber stoppers and total volume of 250 mL of NMS medium. Each incubation was inoculated with the same amount of concentrated enrichment culture, closed and crimp sealed. For abiotic controls (heat killed), the inoculated enrichment co-culture was autoclaved. All bottles (except for the unamended experiments) were supplemented with methane (CH_4_, 99.99% pure) corresponding to a percentage of the headspace volume (0.5, 5, 10%). Time zero samples were also taken at the start of each experiment and filtered onto muffled glass fiber filters (GF/F 47 mm diameter with 0.3 μm pore size; Whatman) and frozen at −80°C until analysis. The bottles were then shaken for 10 s to establish an equilibrium between the gas and water phase. The resulting CH_4_ concentration in the headspace was measured by piercing the butyl septum with a syringe to retrieve a gas sample that was analyzed with gas chromatography-flame ionization detection (GC-FID; Thermo Scientific Focus GC). The bottles were incubated under oxic conditions in the dark at 15°C unless other temperatures are specified. For the salinity experiments, the initial enrichment co-culture was gradually adapted to higher sodium chloride (NaCl) additions to NMS media over a period of 6 months. The resulting experiments were conducted as previously described with unamended controls (CH_4_ 0%). Methane concentrations in the headspace were regularly monitored throughout the experiment. The incubations were ended when the CH_4_ concentration in the headspace was <10% of the initial CH_4_ concentration, and incubations reached a stationary phase based on the reduced rate of decrease in CH_4_ concentrations. The incubations lasted from 6 days to 48 days depending on the growth conditions. The methane oxidation rates for each individual treatment were calculated from fitted slopes spanning the linear phase of methane removal after the initial lag phase. Experiments were ended by filtering the cultures onto muffled glass fiber filters (GF/F 47 mm diameter with 0.3 μm pore size; Whatman) and were immediately frozen at −80°C until lipid and/or DNA extractions.

### 2.2 Methylotenera mobilis

Biomass of *Methylotenera mobilis* (DSM 17540) from the Deutsche Sammlung von Mikroorganismen und Zellkulturen (DSMZ) was previously analyzed for intact polar lipids (Richter et al., 2023). Existing UHPLC-HRMS data was used in this study for the identification of quinones.

### 2.3 Methylovulum psychrotolerans

*Methylovulum psychrotolerans* (Sph56, NCBI Accession number MH701868) was grown in NMS media at different temperatures (4, 10, and 20°C) as described by Bale et al. (2019). Existing UHPLC-HRMS data for intact polar lipids was used for the identification of BHPs and quinones in this study.

### 2.4 Lipid extractions

All filters from the *Methylobacter-Methylotenera* enrichments were divided in half, freeze-dried, and extracted using a modified Bligh-Dyer method (Bligh and Dyer, 1959; Bale et al., 2021). The filters were ultrasonically extracted twice using methanol (MeOH), dichloromethane (DCM), and phosphate buffer (2:1:0.8, v:v:v). DCM and phosphate buffer was added to the resulting solvent in a separate flask for a new volume ratio of 1:1:0.9 (v:v:v). The DCM layer was collected and the remaining aqueous layer was washed twice using DCM. The filters were ultrasonically extracted two more times using MeOH:DCM:aqueous trichloroacetic acid (TCA) solution (2:1:0.8, v:v:v). The same procedure as described above was used to collect the DCM layers. The combined DCM layers were then dried using N_2_ gas and stored at −20°C. Before analysis, an internal standard (deuterated diacylglyceryltrimethylhomoserine; DGTS-d9; Avanti^®^ Polar Lipids, United States) was added to the total lipid extracts (TLEs). The samples were dissolved in MeOH:DCM (9:1, v:v) and filtered through 0.45 μm regenerated cellulose syringe filter (4 mm diameter, Grace Alltech, Deerfield, IL).

### 2.5 Lipid analysis

TLEs for the *Methylobacter-Methylotenera* incubation experiments were analyzed after Hopmans et al. (2021) to identify BHPs. All samples were analyzed on an Agilent 1290 Infinity I UHPLC coupled to a quadrupole-orbitrap (Q-Exactive) HRMS equipped with an Ion Max source and heated electrospray ionization (HESI) probe (ThermoFisher Scientific, Waltham, MA). An Acquity C18 BEH column (2.1 × 150 mm, 1.7 μm particle; Waters) and pre-column was used for separation with a solvent system of (A) MeOH:H_2_O (85:15) and (B) MeOH:isopropanol (1:1) containing 0.12% (v/v) formic acid and 0.04% (v/v) aqueous ammonia in both solvents. A positive ion monitoring mode of *m/z* 350–2,000 (resolution 70,000 ppm at *m/z* 200) and an inclusion list of 357 calculated exact masses of BHPs was used for lipid detection. We used a data dependent MS^2^ with an isolation window 1 *m/z*; resolution 17,500 ppm at *m/z* 200 of the 10 most abundant ions for a total cycle of ca. 1.2 s and dynamic exclusion (6 s) with a 3 ppm mass tolerance to identify BHPs. A stepped normalized collision energy of 22.5 and 40 was used for optimal fragmentation of BHPs. Every 48 h a mass calibration using a Thermo Scientific Pierce LTQ Velos ESI Positive Ion Calibration Solution was performed. BHPs for *Methylovulum psychrotolerans* (Sph56) and quinones for all samples were analyzed using the same method as described above, but with a stepped normalized collision energy of 15, 22.5, and 30 (Bale et al., 2019).

All peak areas were corrected for matrix effects and variability in MS performance with an internal standard, DGTS-d9. Authentic standards to determine absolute BHP and quinone concentrations currently do not exist, therefore all lipids are reported using their relative peak area as response units (RU). Lipids from the co-culture enrichments and the *Methylotenera mobilis* biomass sample are additionally normalized to liters of media filtered and grams of freeze-dried biomass, respectively. Therefore, all reported lipid concentrations are expressed as response units per liter (RU/L) or response units per gram (RU/g).

### 2.6 DNA extraction and 16S rRNA gene amplicon sequencing

A subset of samples was extracted for DNA to confirm that *Methylobacter* sp. and *Methylotenera* sp. were the primary bacteria present in the enrichment culture and that there were no major changes in its composition under different incubation conditions. Time 0 samples were taken at the start of the different experimental set ups and extracted for DNA. Triplicate incubations that were grown under standard conditions (CH_4_ 5%, 15°C, 0 g/L NaCl in NMS media and in the dark), as well as triplicate incubations with large changes in lipid composition (i.e., temperature 30°C and 10 g/L NaCl addition) were also extracted.

A quarter of the filters were extracted for DNA using the DNeasy PowerSoil Pro Kit (Qiagen). A negative extraction blank and mock culture sample (ZymoBIOMICS^®^ Gut Microbiome Standard, Zymo Research Copr.) were included in the extractions as negative and positive controls, respectively. The universal (bacterial and archaeal) primer pairs, 515F and 806RB, were used to target the V4 region of the small subunit ribosomal RNA region (Caporaso et al., 2011; Apprill et al., 2015; Parada et al., 2016). PCR reactions were performed in Phusion buffer (Qiagen) with dNTPs and InvitrogenTM PlatinumTM SuperFiTM Polymerase (Thermo) with the following conditions: 98°C for 30 s, 98°C for 10 s, 30 cycles of 98°C for 10 s, 50°C for 15 s, and 72°C for 30 s, followed by 72°C for 5 s, and 4°C for 5 s. The resulting PCR products were pooled in equimolar amounts and loaded on a 1% agarose gel. The target bands were cut out and purified using QIAquick^®^ PCR gel extraction kit (Qiagen). Samples were sent to the University of Utrecht Sequencing Facility (USEQ, the Netherlands) for Truseq DNA nano library preparation and sequencing on an Illumina NextSeq2000 (Illumina, San Diego, CA) 2 × 300 bp sequencing platform. All sequences are available at the sequence read archive under the BioProject PRJNA1149959.

16S rRNA gene amplicon sequences were analyzed using the Cascabel pipeline (Abdala Asbun et al., 2020). This included quality assessment using FastQC (Andrews, 2010), paired-end reads assembly with PEAR (Zhang et al., 2014), library demultiplexing using QIIME (Caporaso et al., 2010), and picking and taxonomy assignment of amplicon sequence variants (ASVs) using DADA2 (Callahan et al., 2016). Taxonomy was assigned using Silva 138.1 as a reference database (Quast et al., 2013; Yilmaz et al., 2014).

### 2.7 Statistical analyses

We applied a Hellinger transformation and scaling to the BHP dataset prior to performing a principal component analysis (PCA) to determine what drives the largest variations in BHPs under different treatments for the *Methylobacter-Methylotenera* co-culture experiments. All analyses were performed in R (version 4.3.2; R Core Team, 2023) using the vegan package (version 2.5–7; Oksanen et al., 2022), ggplot2 (version 3.4.0; Wickham et al., 2023), FactoMineR (version 2.7; Lê et al., 2008), and factoextra (version 1.0.7; Kassambara and Mundt, 2020). Methane oxidation rates in the experimental conditions were statistically compared to those of the unamended and heat killed incubations using a one-way ANOVA test and Tukeys Honest Significant Differences (HSD).

## 3 Results

### 3.1 Methane-oxidation rates in *Methylobacter-Methylotenera* enrichment incubations

**Figure 1:**
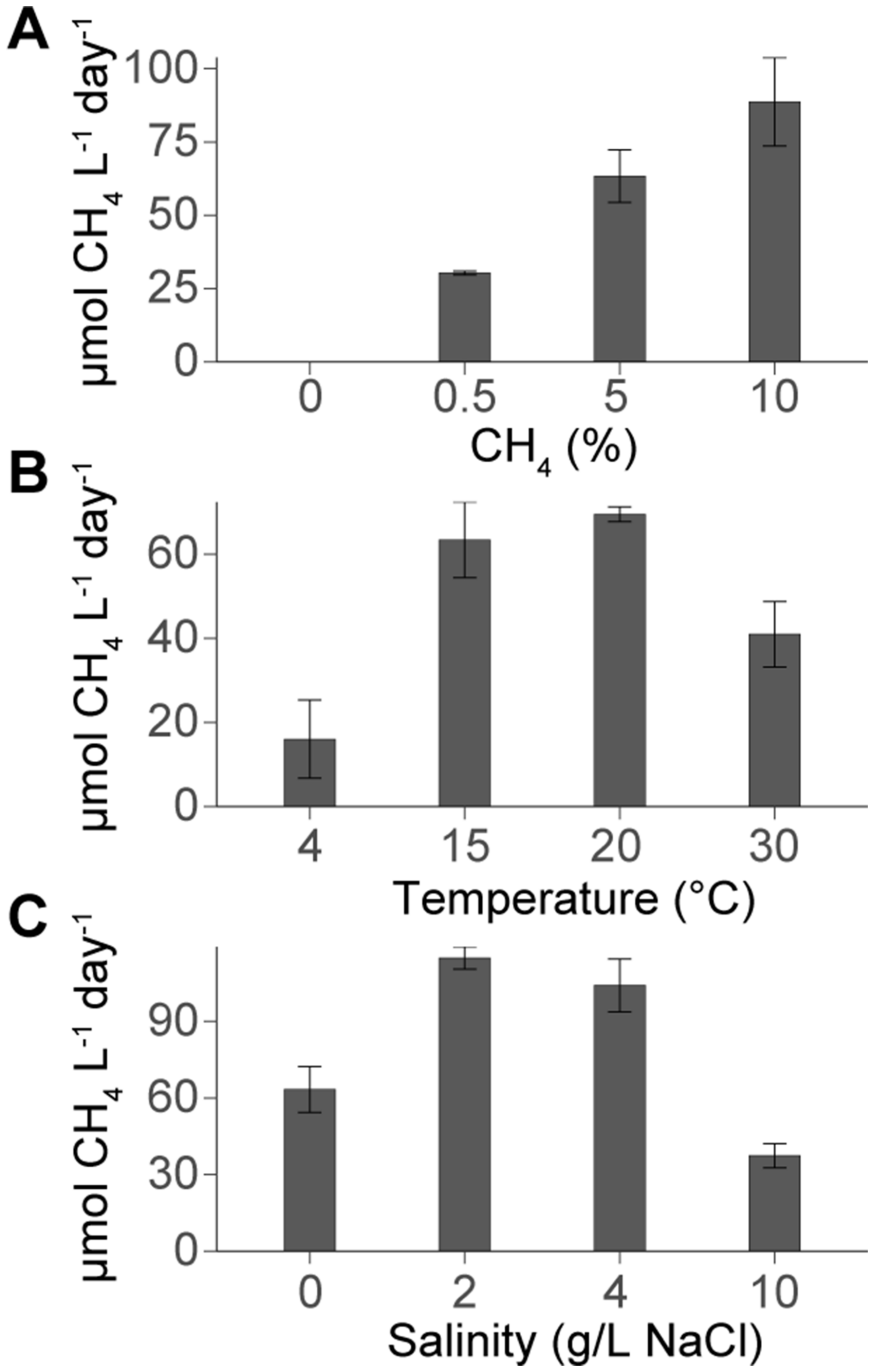
Average methane oxidation rates (μmol CH_4_ L^−1^ day^−1^) and standard deviations (where *n* = 3) for *Methylobacter-Methylotenera* experimental set ups. Experiments were grown with **(A)** different methane concentrations (expressed by the amount of methane (%) added to the headspace), **(B)** temperatures, and **(C)** variations in salinities. At standard conditions, the *Methylobacter-Methylotenera* enrichment was amended with 5% CH_4_ and grown at 15°C with 0 g/L NaCl. For each experimental set up, only one parameter was varied (i.e., methane concentrations, temperature, or salinity) while the other parameters remained constant. Methane oxidation rates were corrected using heat killed controls (see Supplementary Table S1 for all methane oxidation rates and experimental conditions).

*Methylobacter-Methylotenera* enrichments were grown in triplicate under different methane concentrations, temperatures, and salinities. Methane concentrations in the headspace were regularly measured until the enrichment reached the stationary phase when the rate of methane consumption slowed. The resulting methane-oxidation rates were calculated over the period of maximum methane consumption. The methane-oxidation rates for the experimental conditions were corrected using the heat killed controls to account for any potential loss of methane during the experiments through the rubber stoppers (Figure 1; Supplementary Table S1). Methane-oxidation rates in the unamended controls were close to zero, indicating there was no methane production or consumption without the addition of methane. In general, we observe the highest methane-oxidation rates at 10% methane concentrations, temperatures of 15 and 20°C, and lower salinities (0–4 g/L NaCl).

### 3.2 Verification of the *Methylobacter-Methylotenera* enrichment culture composition

The original co-culture of *Methylobacter* sp. and *Methylotenera* sp. was enriched from a lake (van Grinsven et al., 2020). The 16S rRNA gene amplicon results confirmed that *Methylobacter* spp. remains the primary methanotroph present in the time 0 samples (i.e., representing 79% of the total 16S rRNA gene reads; Table 1) and the incubations grown at 5% CH_4_ and 15°C with 0 g/L NaCl (84% of the total 16S rRNA gene reads). However, the relative abundance of *Methylobacter* spp. decreased in the incubations grown at 30°C (61% of the total 16S rRNA gene reads) and at 10 g/L NaCl (63% of the total 16S rRNA gene reads). *Methylomonas* spp. was the only other methanotroph detected but was present in low abundance (0.1% of the total 16S rRNA gene reads) at standard conditions (i.e., 5% CH_4_ and 15°C with 0 g/L NaCl). The methylotroph, *Methylotenera* spp., increased in relative abundance at 30°C (26% of the total 16S rRNA gene reads), but decreased at higher salinities (0.1% of the total 16S rRNA gene reads). *Methylophilus* spp., a methanol-utilizing bacteria, increased in relative abundance (15% of the total 16S rRNA gene reads) at 10 g/L of NaCl relative to experiments with 0 g/L of NaCl (0.3% of the total 16S rRNA gene reads). In addition, 16S rRNA gene sequences attributed to other non-methanotrophs increased in the experiments grown with 10 g/L of NaCl (22% of the total 16S rRNA gene reads) relative to the experiments grown with 0 g/L of NaCl (10% of the total 16S rRNA gene reads) with the most abundant 16S rRNA gene reads being attributed to the families: *Chitinophagaceae*, *Flavobacteriaceae*, *Devosiaceae*, and *Optiutaceae*.

**Table 1:** Results from 16S rRNA gene amplicon sequencing reported as average relative abundance (% of total) and standard deviation (*n* = 3) of 16S rRNA gene reads for methanotrophs and methylotrophs detected in the *Methylobacter-Methylotenera* enrichment (where Temp. = temperature and n.d. = not detected).

Growth conditions				Relative abundance of 16S rRNA gene reads (% of total)				
Experiment	CH_4_ (%)	Temp. (°C)	Salinity (g/L NaCl)	*Methylobacter* spp.	*Methylomonas* spp.	*Methylotenera* spp.	*Methylophilus* spp.	Other
Time 0*	–	15	0	78.8 ± 2.3	0.1 ± 0.1	9.4 ± 2.5	1.2 ± 1.2	10.5 ± 2.2
Methane	5	15	0	83.6 ± 0.9	0.1 ± 0.1	2.6 ± 0.4	0.3 ± 0.1	13.3 ± 0.7
Temp.	5	30	0	60.7 ± 5.6	n.d.	25.6 ± 2.6	2.6 ± 1.1	11.0 ± 3.5
Salinity	5	15	10	62.9 ± 5.4	n.d.	0.1 ± 0.0	15.3 ± 3.3	21.7 ± 2.2

Total reads per sample are reported in Supplementary Table S2.

*Time 0 samples were grown under standard conditions (15°C with 5% CH_4_ and 0 g/L NaCl) and taken at the start of each experiment except for the salinity cultures, as these were slowly adapted to higher salinities over several months and time 0 samples could not be grown at standard conditions.

### 3.3 Variations in respiratory quinone distributions

**Figure 2:**
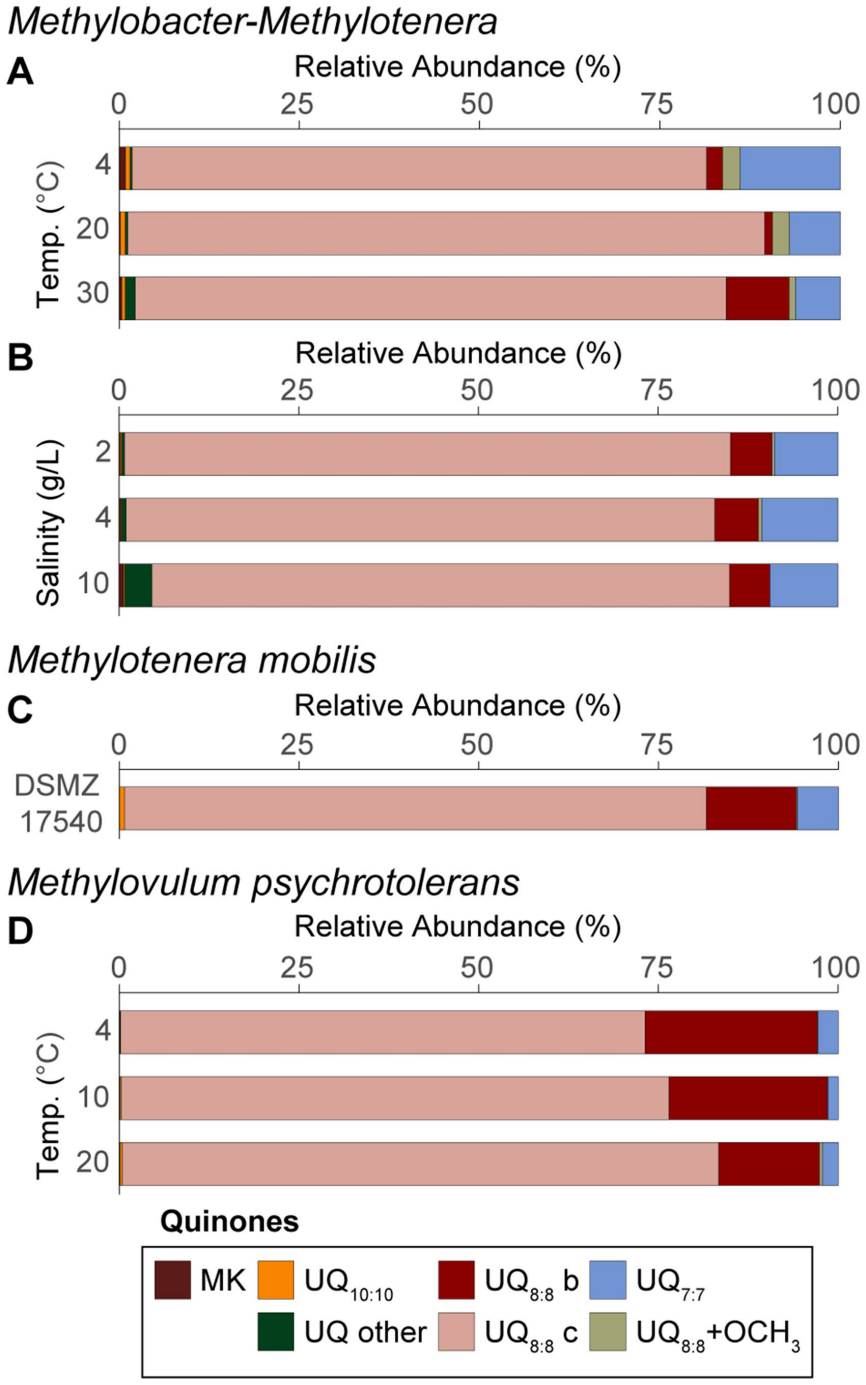
Quinone distributions from the *Methylobacter-Methylotenera* enrichment for the **(A)** temperature experiments and **(B)** salinity enrichments. Quinone distributions are also shown for **(C)** biomass from *Methylotenera mobilis* (DSMZ 17540) and **(D)** *Methylovulum psychrotolerans* grown at different temperatures where UQ = ubiquinone and MK = menaquinone, and where “b” and “c” refer to isomers. Quinone isomers were named as “a,” “b,” and “c” based on the retention times for all quinones identified in the three cultures. UQ_7:7_, UQ_8:8_ + OCH_3_, UQ_10:10_ represent sums of all the isomers. “UQ other” is a sum of all other ubiquinones detected and “MK” is a sum of all menaquinones detected. See Supplementary Tables S3–S5 for the full list of respiratory quinones. Panels **(A,B)** represent an average of triplicate experiments. Triplicates were not available for panels **(C,D)** and reflect single experiments. Also, note that in *Methylobacter-Methylotenera* enrichments grown at temperatures of 15°C and a salinity of 0 g/L NaCl with 0, 5, and 10% methane additions quinones were not detected and therefore are not included in this figure.

Respiratory quinone distributions for the *Methylobacter-Methylotenera* co-culture experiments are shown as averages of the triplicate incubations grown at different temperatures (Figure 2A) and salinities (Figure 2B). Note, that respiratory quinones were not detected in *Methylobacter-Methylotenera* enrichments grown with 0, 5, and 10% methane additions at temperatures of 15°C and salinities of 0 g/L NaCl. We speculate this might be related to the lipid extraction procedure, but we are unsure why this was the case. To determine which respiratory quinones are likely being produced by *Methylotenera* spp. in the enrichment experiments, the quinone distributions of a pure culture from a closely related strain to that of *Methylotenera* sp. in the enrichment, i.e., *Methylotenera mobilis*, were also analyzed (Figure 2C).

To evaluate whether methanotrophs adapted to extreme environmental conditions have a similar metabolic response to environmental stress, we also report changes in respiratory quinones for a psychrotolerant methanotroph, *Methylovulum psychrotolerans*, grown at different temperatures (Figure 2D). Note, *Methylovulum psychrotolerans* were not grown in triplicate, so lipid results reflect single incubation experiments.

In all cultures, ubiquinones with 8 isoprenoid units and 8 unsaturations (UQ_8:8_), followed by UQ_7:7_ were the most abundant quinones. In the *Methylobacter-Methylotenera* enrichment, methylene-ubiquinone (MQ_8:7_) was also detected. A UQ_8:8_ with an additional methoxy group on the unsaturated isoprenoidal chain (UQ_8:8_ + OCH_3_) was identified in both the enrichment and *Methylotenera mobilis*. Further, a UQ_8:8_ with an additional methoxy group and additional hydroxyl group on the side chain (UQ_8:8_ + OCH_3_ + OH) was observed in the enrichment (see Supplementary material S3.2). UQ_9:9_ and UQ_10:10_ were identified in all samples, but in low relative abundance. Menaquinones (i.e., MK_6:6_, MK_7:7_, and MK_8:8_) were only found in the *Methylobacter-Methylotenera* enrichment incubations, but in minor proportions (i.e., <1%). The full list of quinones identified in the *Methylobacter-Methylotenera* enrichments, *Methylotenera mobilis*, and *Methylovulum psychrotolerans* are listed in Supplementary Tables S3–S5.

### 3.4 BHP distributions in methanotroph culture experiments

**Figure 3:**
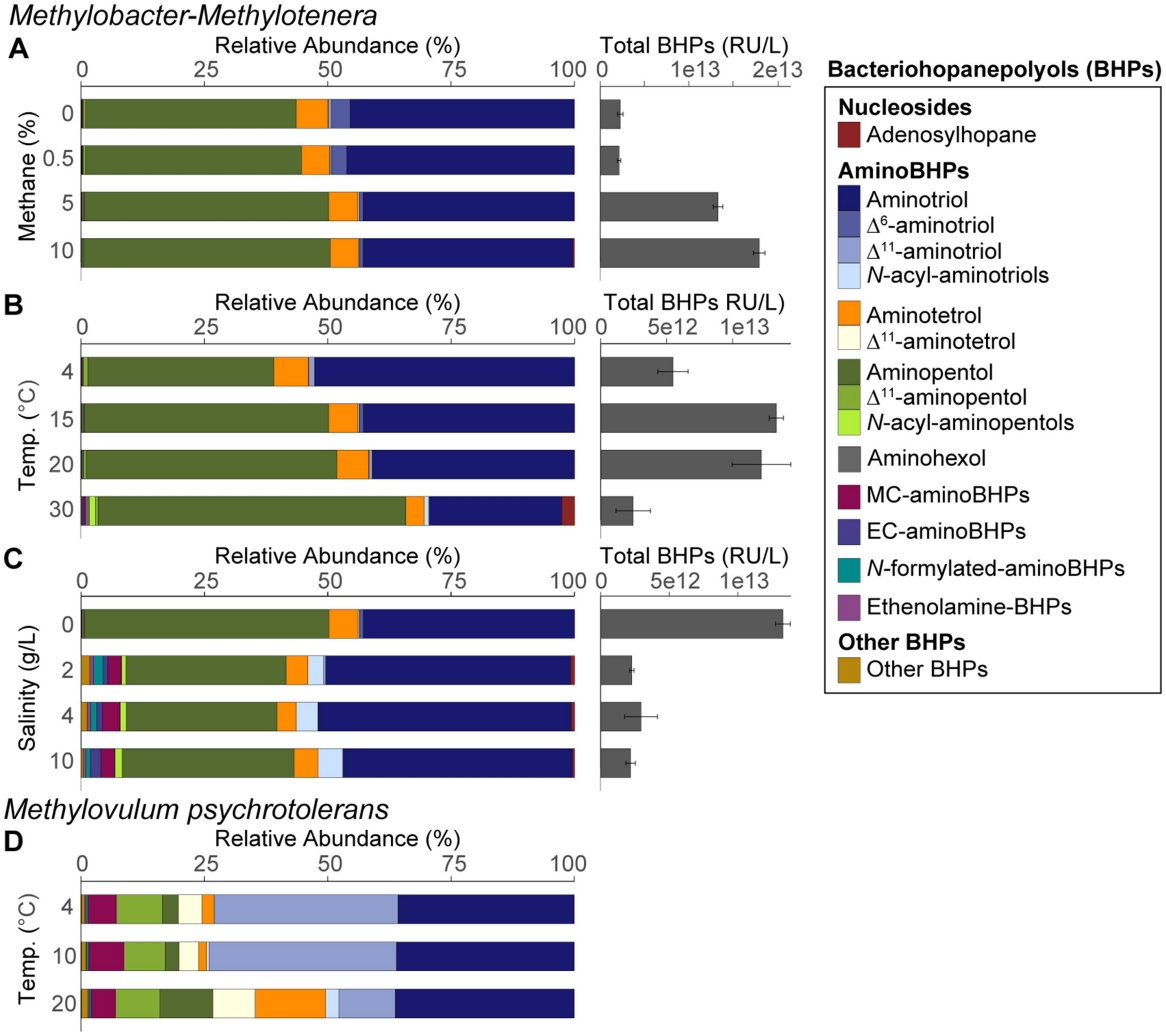
BHP distributions and total BHP concentrations (response units per liters) for the *Methylobacter-Methylotenera* enrichment for **(A)** methane concentration experiments, **(B)** different temperature treatments, and **(C)** salinity enrichments. Panels **(A–C)** are averages of triplicate experiments. BHP distributions are also shown for **(D)** *Methylovulum psychrotolerans* that reflects single culture experiments where unsat. = unsaturated, MC = methylcarbamate, and EC = ethylcarbamate. *N*-acyl-aminotriols, *N*-acyl-aminopentols, aminohexols, MC-aminoBHPs, EC-aminoBHPs, *N*-formylated-aminoBHPs, and ethenolamine-BHPs represents a sum of BHPs with similar functional groups. “Other BHPs” is a sum of all other BHPs that were detected in low abundance, including unknown composite BHPs. See Supplementary Tables S5, S6 for the full list of BHPs.

BHP distributions are reported as averages of triplicates for the *Methylobacter-Methylotenera* enrichment grown under different methane concentrations, temperatures, and salinities (Figures 3A–C). *Methylotenera mobilis* (DSM 17540), a phylogenetically closely related species of the methylotroph identified in the enrichment co-culture, was previously analyzed to confirm that the methylotroph does not produce any BHPs (Richter et al., 2023). The BHP distributions for *Methylovulum psychrotolerans* grown under different temperatures are also reported (Figure 3D). All the BHPs identified in this study are listed in Supplementary Tables S6, S7.

**Figure 4:**
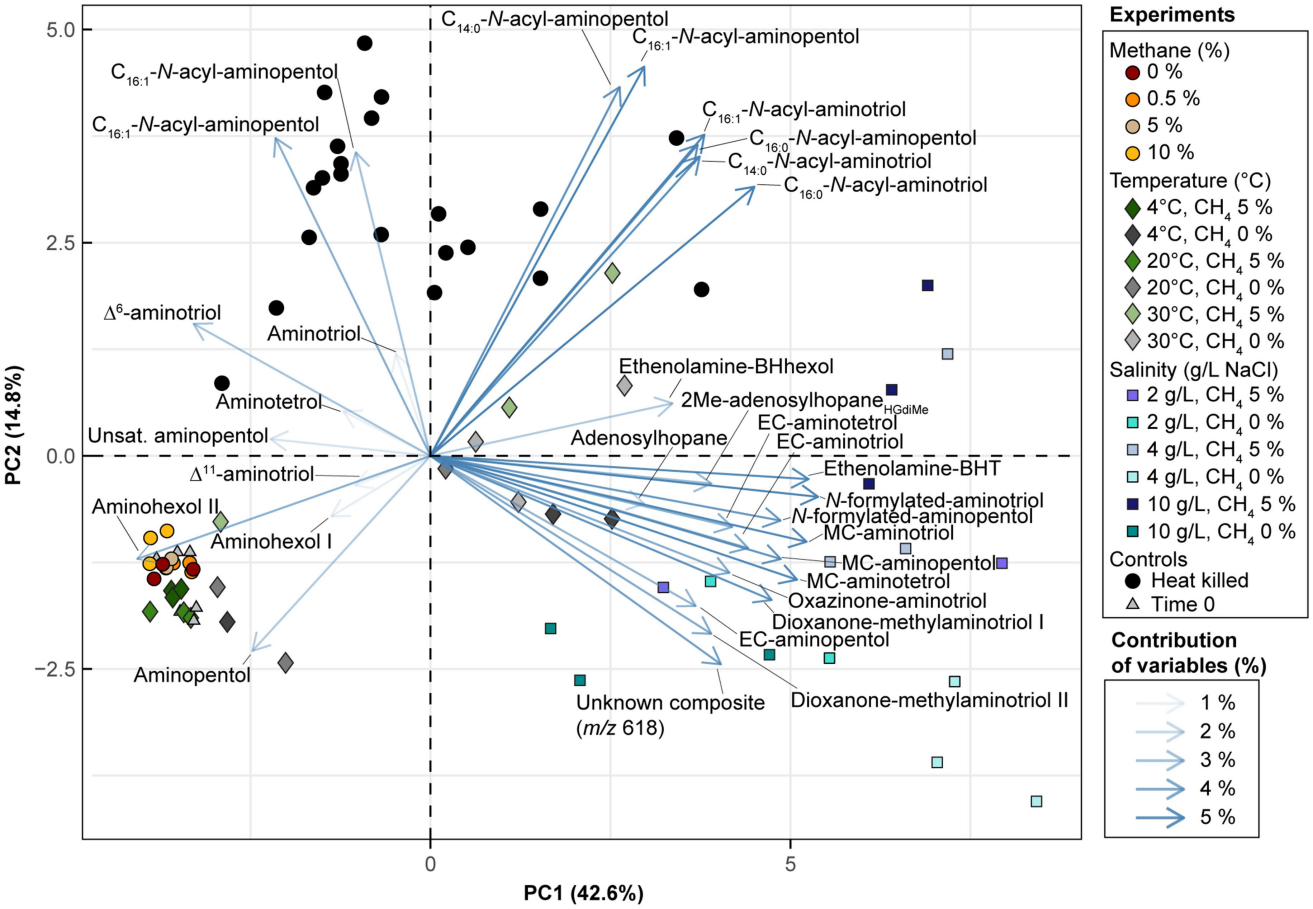
Biplot visualization of PC1 and PC2 for the BHP dataset from the *Methylobacter-Methylotenera* enrichment culture. The experiments are colored by treatment type (for details see Supplementary Table S1). The BHP loadings are colored by their average contribution to PC1 and PC2 where unsat. = unsaturated (unknown double bond position), MC = methylcarbamate, and EC = ethylcarbamate.

In the *Methylobacter-Methylotenera* enrichment experiments, 35-aminobacteriohopane-32,33,34-triol (aminotriol from herein) and aminopentol were the most abundant BHPs (Figures 3A–C). The PCA of the BHP data confirms that the BHPs extracted from our triplicates were consistent with each other and cluster together in the biplot, with the exception of one of the triplicates grown at a temperature of 30°C (Figure 4). Further, this highlights that the largest variability observed in the BHP distributions occurred in incubations grown at higher salinities (i.e., PC1 42.6%).

Using new analytical techniques, we were able to detect a much broader diversity of BHPs in our samples (Hopmans et al., 2021; Figures 3A–C) than observed using previous analytical techniques (Schulenberg-Schell et al., 1989; Moreau et al., 1995; Talbot et al., 2001, 2016; Kusch et al., 2018). Notably, BHPs with an ethyl group attached to the carbamate moiety of the side chain were detected in the *Methylobacter-Methylotenera* enrichments grown at higher salinities (Figure 3C; Supplementary Figure S8). We refer to these BHPs as ethylcarbamate (EC)-aminoBHPs: EC-aminotriol, EC-aminotetrol, and EC-aminopentol (see Supplementary material S4.2.1). In addition, an unknown composite BHP was also detected in *Methylobacter-Methylotenera* enrichments adapted to higher salinities (Supplementary material S4.2.2; Supplementary Figure S7).

The most abundant BHPs detected in *Methylovulum psychrotolerans* were aminotriol and Δ^11^-aminotriol, followed by aminotetrol, aminopentol, and their unsaturated versions (Figure 3D). In addition, methylcarbamate-aminoBHPs, ethenolamine-BHPs, *N*-formylated-aminoBHPs, and a novel unknown composite BHP were also detected in the *Methylovulum psychrotolerans* cultures (Supplementary material S4.2.3; Supplementary Figure S8).

## 4 Discussion

### 4.1 Lipid response of *Methylobacter-Methylotenera* to increasing methane concentrations

In the *Methylobacter-Methylotenera* enrichment experiments, methane oxidation rates increased with increasing methane concentrations (Figure 1). In good agreement with these results, methane concentrations in lakes are usually positively correlated with methane oxidation rates (Kankaala et al., 2006; Guérin and Abril, 2007; Martinez-Cruz et al., 2015), suggesting that methanotrophs are sensitive to changes in methane availability and thereby actively regulate the amount of methane emitted from lakes.

The 16S rRNA gene amplicon sequencing results showed that *Methylobacter* spp. was the most abundant methanotroph in the 5% CH_4_ amended experiments (83% of the total 16S rRNA gene reads; Table 1). The BHP distributions of all methane amended enrichment experiments are characterized by a high abundance of aminotriol and aminopentol (Figure 3A); this is consistent with the BHP distributions observed in other *Methylobacter* strains (Osborne, 2015; Rush et al., 2016). We observed no major changes in the overall BHP distributions in the 0, 0.5, 5, and 10% CH_4_ amended enrichment experiments (Figure 3A). This is confirmed by a PCA, where the methane concentration experiments all clustered together with our time zero samples (Figure 4). Overall, these results point to the utility of aminotriol and aminopentol as chemotaxonomic biomarkers for *Methylobacter* methanotrophs, which are an important component of the methanotrophic community contributing to methane oxidation in freshwater systems (Knief, 2015).

The total BHP concentrations in the enrichments (not corrected for cell density) increased with increasing CH_4_ and higher methane oxidation rates, which reflects an increase in aminotriol, aminotetrol, aminopentol in the CH_4_ amended experiments relative to the unamended CH_4_ experiments (Figure 3A). In previous enrichment incubations for methanotrophs, only aminopentol concentrations increased with increasing methane concentrations, likely reflecting increased growth or activity of *Methylobacter* sp. present in the microcosm experiments (Osborne, 2015; Sherry et al., 2016). An increase in aminoBHP abundance is often interpreted as evidence for increased methane-oxidation by MOB in the environment, particularly in paleo-records (Coolen et al., 2008; Blumenberg et al., 2013; Talbot et al., 2014). Further work with pure MOB cultures and exact concentrations of BHPs per cell are needed to confirm whether the increase in the total absolute abundance of aminoBHPs at higher methane concentrations reflects an increase in cells oxidizing methane or a higher concentration of aminoBHPs in a small percentage of cells performing methane oxidation.

### 4.2 Methanotroph lipid response to temperature

The highest methane oxidation rates for the *Methylobacter-Methylotenera* enrichments occurred at 15 and 20°C (Figure 1). This reflects the optimal growth temperatures for the mesophilic members of the *Methylobacter* and *Methylotenera* genus at 23–35°C and 18–21°C, respectively (Afshin et al., 2021; Collins et al., 2017). *Methylovulum psychrotolerans* is considered psychrotolerant and can grow at temperatures from 2 to 36°C, but with optimal growth also occurring between 20 and 25°C (Oshkin et al., 2016).

*Methylovulum psychrotolerans* and *Methylotenera mobilis* both have a similar quinone distribution to that of the *Methylobacter-Methylotenera* enrichment, where UQ_8:8_ is the most abundant quinone, followed by UQ_7:7_ (Figure 2). Proteobacteria, including Betaproteobacteria and Gammaproteobacteria, are known to produce UQ_8:8_ and UQ_7:7_ in high abundance (Collins and Green, 1985; Hiraishi, 1999; Kersters et al., 2006). In *Methylovulum psychrotolerans,* the relative abundance of UQ_8:8_ increases with temperature from 4 to 20°C. Similarly, the relative abundance of UQ_8:8_ increases at 20°C in the *Methylobacter-Methylotenera* co-culture compared to the incubations at 4 and 30°C (Figure 2A). Increased relative abundance of UQ_8:8_ at 20°C suggests that production of this quinone is associated with optimal growth conditions due to either an increase in biomass or an increase in UQ_8:8_ production per cell. In the *Methylobacter-Methylotenera* enrichment both menaquinones and UQ_8:8_ with methoxy groups increase at lower temperatures (Figure 2A); however, this does not occur in *Methylovulum psychrotolerans*. It was previously demonstrated that in *Listeria moncytogenes* the menaquinone content in the cell membrane increases at lower temperatures to improve membrane fluidity (Seel et al., 2018; Flegler et al., 2021). This might also explain the increase in menaquinones in the *Methylobacter-Methylotenera* enrichments. Alternatively, the changes in quinone distributions observed in the *Methylobacter-Methylotenera* enrichment could be attributed to a shift in the microbial community composition at lower temperatures.

In the *Methylobacter-Methylotenera* enrichment, *Methylobacter* spp. is the most abundant methanotroph in the 15 and 30°C temperature experiments (Table 1) and also the primary BHP producer, as *Methylotenera* spp. does not produce BHPs (Richter et al., 2023). In addition, all other species detected in the culture are present in low relative abundance (Table 1). In the BHP distributions, aminotriol decreases at higher temperatures, whereas the relative abundances of aminopentol and adenosylhopane both increase (Figure 3C). In the Type I methanotroph, *Methylovulum psychrotolerans*, the relative abundance of aminotetrol and aminopentol increase with temperature from 4 to 20°C (Figure 3D). A similar increase in aminoBHPs at higher temperatures was previously observed in the River Tyne microcosm experiments where the highest concentrations of aminotriol and aminotetrol occurred between 4 and 21°C and aminopentol increased from 4 to 40°C (Osborne et al., 2017). However, community succession of different species of *Methylobacter* sp. in the River Tyne experiments explained some of the differences in aminoBHP production at different temperatures (Sherry et al., 2016; Osborne et al., 2017). Pure culture experiments with another Type I methanotroph (strain CEL 1923) did not show the same trends observed in our cultures, where only aminotetrol increased with temperature and aminopentol decreased at higher temperatures (Jahnke et al., 1999). This could indicate species-specific adaptations of BHP production at different temperatures. In mesocosm experiments in *Sphagnum* peat bogs that were likely dominated by Type II methanotrophs, aminotriol, aminotetrol, and aminopentol concentrations also increased in response to increasing temperatures from 5 to 25°C (van Winden et al., 2020). These studies suggest that changes in aminoBHPs could reflect either community succession or a physiological adaptation at different temperatures. In the case of the *Methylobacter-Methylotenera* enrichment and in *Methylovulum psychrotolerans*, the increase in aminotetrol and aminopentol reflects an increase in hydroxyl groups likely promoting increased hydrophilic interactions among lipids in the lipid membrane. An increase in the number of hydroxylations at higher growth temperatures was previously observed in membrane lipids in *Rhizobium tropici* and *Burkholderia cepacian* to increase the lateral interactions between lipid molecules in response to stress (Taylor et al., 1998; Vences-Guzmán et al., 2011). Thus, an increase in aminoBHPs with more hydroxyl groups could be a stress related response in the *Methylobacter-Methylotenera* enrichment and in *Methylovulum psychrotolerans*.

PCA results from the *Methylobacter-Methylotenera* enrichment experiments show that Δ^6^- and Δ^11^-aminotriol have a minor contribution to temperatures below 30°C (Figure 4). We observed a slight increase in Δ^6^-aminotriol at lower temperatures. However, this response in the *Methylobacter-Methylotenera* enrichment was much smaller than that observed in *Methylovulum psychrotolerans*, where Δ^11^-aminotriol, Δ^11^-aminotetrol, Δ^11^-aminopentol, unsat. MC-aminotriol, unsat. ethenolamine-bacteriohopanetetrol, and unsat. *N*-formylated-aminotriol all increase with decreasing temperatures relative to their saturated counterparts (Figure 3D). So far, the increase in unsaturated BHPs at colder temperatures has not been observed in other methanotroph cultures (Jahnke et al., 1999; Osborne et al., 2017; van Winden et al., 2020). In the River Tyne enrichment experiments, for instance, unsaturated BHPs were only observed at high temperatures (i.e., 40 and 50°C) and corresponded to a shift in the methanotroph community to a different species of *Methylobacter* and the thermophilic genus *Methylocaldum* (Sherry et al., 2016; Osborne et al., 2017). This suggests that mono-unsaturated aminoBHPs, in particular Δ^11^-aminopentol, could be a unique temperature adaptation in certain methanotrophs, such as *Methylovulum* spp. and *Methylocaldum* spp., for maintaining membrane homeostasis under colder conditions (Cvejic et al., 2000; van Winden et al., 2012; Bale et al., 2019). The differences in BHP response to temperature in the *Methylobacter-Methylotenera* enrichment compared to *Methylovulum psychrotolerans* and previous culture studies (i.e., Jahnke et al., 1999; Osborne et al., 2017; van Winden et al., 2020) suggests species specific adaptations in BHPs to changes in temperature. In general, this highlights the need for more environmental and culture studies to understand how temperature affects BHP distributions in MOB.

### 4.3 Salinity effects on methanotroph-associated BHPs and quinones

Adapting the *Methylobacter-Methylotenera* enrichment to different salinities resulted in a notable decrease in methane oxidation rates at 10 g/L NaCl and total BHP concentrations (not corrected for cell density; Figure 1C), likely because this enrichment was originally isolated from a freshwater lake and not accustomed to large changes in salinity (van Grinsven et al., 2020). In these experiments, *Methylobacter* spp. remains the primary methanotroph at 10 g/L NaCl and other methanotrophs present at 0 g/L NaCl are absent from our 16S rRNA gene amplicon results. For the methylotrophs, however, *Methylophilus* spp. increased in abundance, whereas *Methylotenera* spp. is largely absent indicating a change in the microbial interactions (Table 1). In pure culture experiments with a haloalkaliphilic Type I methanotroph, *Methylotuvimicrobium alcaliphilum*, growth rates and hopanoid absolute abundance decreased at higher salinities (Cordova-Gonzalez et al., 2021), suggesting that an increase in salinity could also lead to lower methane oxidation rates and BHP abundance in a *Methylotuvimicrobium-*rich community. Similarly, previous enrichment experiments with River Tyne sediments led to a decrease in methane oxidation rates at higher salinities; however, there was no corresponding decrease in total BHP concentrations (Osborne, 2015; Sherry et al., 2016). This lack of change in BHP concentration in the River Tyne enrichments, could be explained by a shift in the methanotroph community from *Methylobacter* to *Methylomicrobium* spp., which was better adapted to growing at higher salinities (Sherry et al., 2016). These results seem to suggest that methane oxidation rates generally decrease at higher salinities, although it is unclear whether this is driven by a decrease in the number of cells or in methanotroph activity.

In the *Methylobacter-Methylotenera* enrichments, the only notable change in quinone distribution with increasing salinity is an increase in UQ_10:10_. Previous studies demonstrated that UQ_10:10_ is directly involved in regulating membrane permeability and elasticity under osmotic stress in an *Escherichia coli* strain (Eriksson et al., 2019). It is likely that UQ_10:10_ plays a similar role in the enrichment culture. However, the increase in UQ_10:10_ could also be linked to non-methanotrophs at 10 g/L NaCl relative to the enrichments grown at 0 g/L NaCl, as shown by the change in the 16S rRNA gene read distribution and a change in dominance in the methylotroph from *Methylotenera* spp. to *Methylophilus* spp. (Table 1).

Salinity accounts for the largest variability of BHPs in the *Methylobacter-Methylotenera* experiments (PC1 42.6%; Figure 4). We observe an overall decrease in the relative abundance of aminopentol relative to 0 g/L NaCl (Figure 3C). The relative abundances of MC-aminotriol, MC-aminotetrol, and MC-aminopentol, as well as their ethylcarbamate forms all increase at higher salinities (Figure 3C). In contrast, there are no significant changes in individual BHP concentrations in the River Tyne incubations at salinities from 1 to 70 g/L NaCl, and only at 120 g/L NaCl do individual BHP concentrations significantly decrease (Osborne, 2015). Incubations at different salinities with the haloalkaliphilic *Methylotuvimicrobium alcaliphilum*, led to either a decrease or no change in individual BHP concentrations at higher salinities (Cordova-Gonzalez et al., 2021). As we are working with an enrichment co-culture (see van Grinsven et al., 2020), the individual changes in BHP abundances could either be explained as a physiological adaptation by *Methylobacter* or a change in the microbial community. As demonstrated by the 16S rRNA gene sequencing results, the relative abundance of 16S rRNA gene reads of non-methanotrophs, such as *Methylophilus* spp., increase at 10 g/L NaCl in the *Methylobacter-Methylotenera* co-culture (Table 1); suggesting that other microbes might also be responsible for BHP production in the enrichments. A protein blast search (NCBI, National Center for Biotechnology Information) for the squalene-hopene cyclase gene of *Bradyrhizobium japonicum* (accession no. WP_038942977.1) did not yield any hits for *Methylophilus* spp. Although this suggests that the *Methylophilus* spp. present in the enrichment might not be responsible for BHP production in the enrichment, it does not rule out the possibility of BHP production by other microorganisms present. As a physiological adaptation, the increase in BHPs with additional modifications to the sidechain was previously proposed to promote intracellular associations that might lead to lipid raft-like domains and tighter packing of saturated phospholipids in a liquid-ordered phase (Sáenz, 2010). More work, however, is needed to test whether this holds true for BHPs with amine functional groups. To simulate natural methanotroph adaptations to changes in salinity, culture experiments with halophilic methanotrophs and/or enrichments from marine sites are needed, and further modifications in membrane lipids should be evaluated.

## 5 Conclusion

Lipid analyses of *Methylobacter-Methylotenera* enrichment from a freshwater lake and from preliminary experiments with an extremophile, *Methylovulum psychrotolerans*, highlight several distinct membrane lipid responses of MOBs to environmental stress. In the *Methylobacter-Methylotenera* enrichments grown at higher methane concentrations both methane-oxidation rates and total BHP concentrations (not corrected for cell density) increased. The increase in BHPs was driven by an increase in aminoBHPs, which should be explored further as potential biomarkers for methane oxidation. In the enrichments and *Methylovulum psychrotolerans*, the relative abundance of aminopentol increased at higher temperatures. In contrast, the relative abundances of unsaturated BHPs increased in the *Methylovulum psychrotolerans* culture at lower temperatures. For *Methylovulum psychrotolerans* and the *Methylobacter-Methylotenera* enrichment, UQ_8:8_ increased in relative abundance at 20°C, corresponding to the optimal growth temperatures of both cultures. This highlights potential specific BHP adaptations in different methanotrophs to variations in temperature, but a more consistent response in ubiquinone distributions. Our salinity incubations account for the largest variance in the BHP dataset for the *Methylobacter-Methylotenera* enrichment, but only a slight increase in UQ_10:10_ and no other major changes in the quinone distributions. The BHP variability at higher salinities is mainly explained by increases in the relative abundance of aminoBHPs with additional modifications to the side chain (e.g., MC-aminoBHPs, EC-aminoBHPs, and *N*-formylated-aminoBHPs). However, more work is needed to test whether this is an adaptation to changes in salinity by methanotrophs. This work highlights the potential of using a combined biomarker approach by analyzing both respiratory quinones and BHPs to understand how environmental changes influence methanotroph activity and lipid membrane adaptations in modern settings.

## Data Availability

All sequence data generated in this study were deposited in the NCBI sequence read archive (SRA) under the BioProject number PRJNA1149959: https://www.ncbi.nlm.nih.gov/bioproject/PRJNA1149959. All other datasets generated in this study are included in the article/Supplementary material.
