# Supplementary material for: Methanotroph-methylotroph lipid adaptations to changing environmental conditions
Source: Front Microbiol. 2025 Feb 7;16:1532719. doi: 10.3389/fmicb.2025.1532719 (PMC11844350; doi:10.3389/fmicb.2025.1532719)
Supplement: Supplementary file 1 [file Data_Sheet_1.PDF]

Supplementary Material

1 Quinone & bacteriohopanepolyol structures

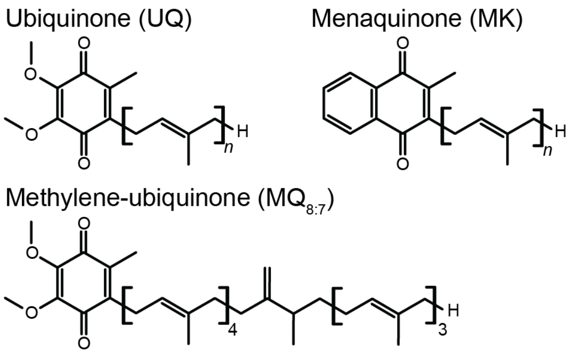

Supplementary Figure 1. Structures of respiratory quinones discussed in this manuscript (see Tables S3-S5 for the full list of quinones).

Nucleosides

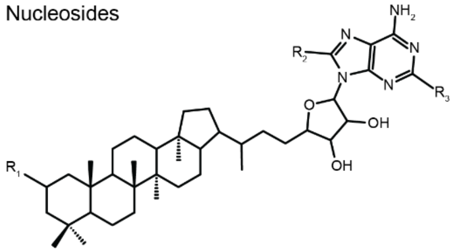

R_1, R_2, and R_3 = H: 30-(5'-adenosyl)hopane ***adenosylhopane***

R_1, R_2, and R_3 = CH_3: ***2Me-adenosylhopane***$_{HG\text{-}diMe}$

35-aminobacteriohopanepolyols

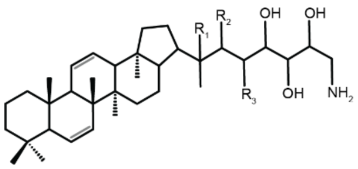

R_1, R_2, and R_3= H or OH

aminotriol, -tetrol, -pentol, and -hexol

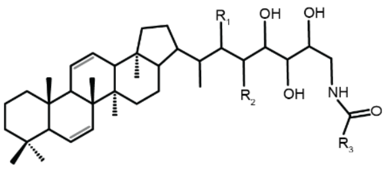

R_1 and R_2= H or OH

R_3 = $C_{13}H_{27}$, $C_{15}H_{31}$, or $C_{15}H_{29}$

C14:0-, C16:0-, and C16:1-N-acyl-aminotriol and -pentol

35-methylcarbamate or ethylcarbamate-aminobacteriohopanepolyols

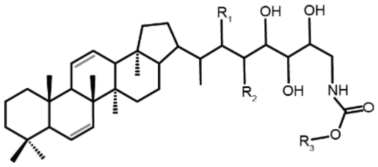

R_1 and R_2= H or OH

R_3= CH_3 or C_2H_5

Methylcarbamate or ethylcarbamate-aminotriol, -tetrol, and -pentol

35-ethenolamine-bacteriohopanepolyols

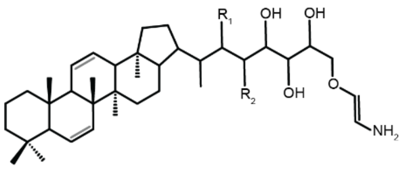

R_1 and R_2= H or OH

Ethenolamine-BHT and -BHhexol

N-formylated-35-aminobacteriohopanepolyols

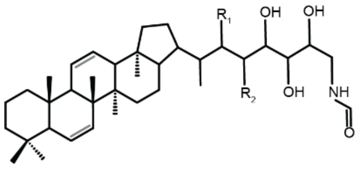

R_1 and R_2= H or OH

N-formylated-aminotriol, -tetrol, and -pentol

Oxazinone- and dioxanone-aminobacteriohopanepolyol

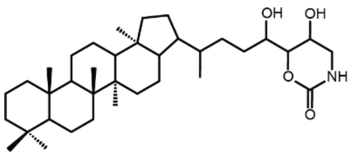

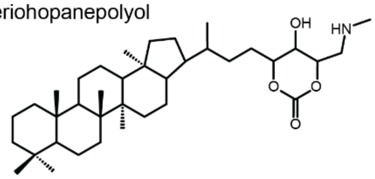

Oxazinone- and dioxanone-aminotriol

Supplementary Figure 2. Structures of all bacteriohopanepolyols (BHPs) discussed in this manuscript (see Tables S6-S7 for the full list of BHPs). The full names for the BHPs are listed, as

well as the names used in this manuscript. The primary core BHP for each group is shown with modifications to the core structure (i.e., unsaturations or methyl groups) and to the side-chain (i.e., hydroxyl groups and alkyl groups) as indicated by R_1, R_2, and R_3.

2 *Methylobacter-Methylotenera* incubations

Supplementary Table 1. Summary of different experimental setups. All experiments were performed in triplicate using nitrate mineral salt (NMS) media where EXP = experimental, HK = heat killed, UN = unamended, and Temp. = temperature. Maximum methane oxidation rates are reported as averages (Avg.) and standard deviation (S.D.) where n = 3. A one-way ANOVA test and Tukey HSD confirms that our EXP setups were significantly different from our HK and UN controls for each condition, i.e. methane concentration (HK-EXP $p < 0.05$), temperature (HK-EXP $p < 0.05$ and UN-EXP $p < 0.001$), and salinity (HK-EXP $p < 0.005$ and UN-EXP $p < 0.005$).

						µmol CH_4 L^{-1} day^{-1}	
**Incubations**	**Experiment**	**Type**	**Temp. (°C)**	**CH_4 (%)**	**Salinity (g/L NaCl)**	**Avg.**	**S.D.**
CH_4 0%	CH_4	EXP	15	0	0	0.01	0.02
CH_4 0% HK	CH_4	HK	15	0	0	0.02	0.04
CH_4 0.5%	CH_4	EXP	15	0.5	0	30.40	1.47
CH_4 0.5% HK	CH_4	HK	15	0.5	0	1.22	1.32
CH_4 5%	CH_4	EXP	15	5	0	104.32	1.83
CH_4 5% HK	CH_4	HK	15	5	0	43.39*	8.81
CH_4 10%	CH_4	EXP	15	10	0	135.84	4.00
CH_4 10% HK	CH_4	HK	15	10	0	50.56*	15.03
Temperature 4°C	Temp.	EXP	4	5	0	40.49	1.78
Temperature 4°C UN	Temp.	UN	4	0	0	0.03	0.02
Temperature 4°C HK	Temp.	HK	4	5	0	25.82*	8.72
Temperature 20°C	Temp.	EXP	20	5	0	100.15	6.00
Temperature 20°C UN	Temp.	UN	20	0	0	0.01	0.01
Temperature 20°C HK	Temp.	HK	20	5	0	32.00*	5.76
Temperature 30°C	Temp.	EXP	30	5	0	69.35	2.47
Temperature 30°C UN	Temp.	UN	30	0	0	0.00	0.00
Temperature 30°C HK	Temp.	HK	30	5	0	27.66*	8.32
Salinity 2g/L NaCl	Salinity	EXP	15	5	2	124.80	8.59
Salinity 2g/L NaCl UN	Salinity	UN	15	0	2	0.01	0.02
Salinity 2g/L NaCl HK**	Salinity	HK	15	5	2	14.22*	7.48
Salinity 4g/L NaCl	Salinity	EXP	15	5	4	107.41	10.46
Salinity 4g/L NaCl UN	Salinity	UN	15	0	4	0.03	0.00
Salinity 4g/L NaCl HK**	Salinity	HK	15	5	4	7.25*	3.29
Salinity 10g/L NaCl	Salinity	EXP	15	5	10	38.18	0.82
Salinity 10g/L NaCl UN	Salinity	UN	15	0	10	0.01	0.01
Salinity 10g/L NaCl HK**	Salinity	HK	15	5	10	2.19*	4.58

*Note: High rates of methane loss in the heat killed controls could result from more brittle rubber stoppers after autoclaving.
**Note: Heat killed controls for salinity were run as negative controls for methane oxidation rates but were not analyzed for BHPs.

Supplementary Table 2. Results from 16S rRNA amplicon sequencing reported as the total reads per sample and the relative abundance (% of total) of 16S rRNA gene reads for methanotrophs and methylotrophs detected in *Methylobacter-Methylotenera* enrichments (where Temp. = temperature and Sal. = salinity).

					Relative abundance of 16S rRNA gene reads (% of total)				
Experiment	CH_4 (%)	Temp. (°C)	Sal. (g/L NaCl)	Total reads	*Methylobacter spp.*	*Methylomonas spp.*	*Methylophilus spp.*	*Methylotenera spp.*	Other
Time 0	-	15	0	677274	81.1	0.1	2.5	7.9	8.4
Time 0	-	15	0	967247	78.7	0.0	0.3	8.1	12.8
Time 0	-	15	0	524008	76.6	0.0	0.9	12.3	10.3
Methane	5	15	0	1722913	84.7	0.1	0.2	2.5	12.5
Methane	5	15	0	215017	82.8	0.0	0.4	3.0	13.8
Methane	5	15	0	372612	83.4	0.2	0.4	2.3	13.6
Temp.	5	30	0	1111746	59.8	0.0	2.1	28.3	9.8
Temp.	5	30	0	696451	55.5	0.0	3.8	25.6	15.0
Temp.	5	30	0	349589	66.6	0.0	1.9	23.0	8.4
Salinity	5	15	10	915434	68.3	0.0	12.4	0.1	19.3
Salinity	5	15	10	761000	57.5	0.0	18.9	0.1	23.5
Salinity	5	15	10	699927	62.9	0.0	14.7	0.1	22.3

3 Respiratory Quinones

3.1 Summary of quinones

Supplementary Table 3. Quinones identified in this study for *Methylobacter-Methylotenera* enrichments where MQ = methylene-ubiquinone, UQ = ubiquinone, and MK = menaquinone. Isomers are indicated as “a”, “b”, and “c” based on retention times for all quinones identified in the three cultures.

Quinone	Isomer	Headgroup	tr (min)	AEC	MS² ion	$M_{calc}(m/z)$	Δppm
$UQ_{8:8}$+OCH_3+OH	a	Ubiquinone	18.00	$C_{50}H_{80}O_6N$	NH_4^+	790.598	-0.50
$UQ_{8:8}$+OCH_3+OH	b	Ubiquinone	18.87	$C_{50}H_{80}O_6N$	NH_4^+	790.598	-0.49
$UQ_{7:7}$	a	Ubiquinone	22.55	$C_{44}H_{67}O_4$	H^+	659.503	-0.38
$UQ_{7:7}$	b	Ubiquinone	24.19	$C_{44}H_{67}O_4$	H^+	659.503	-0.23
$UQ_{8:8}$+OCH_3	a	Ubiquinone	26.56	$C_{50}H_{80}O_5N$	NH_4^+	774.603	-0.92
$UQ_{8:8}$+OCH_3	b	Ubiquinone	27.39	$C_{50}H_{80}O_5N$	NH_4^+	774.603	0.16
$UQ_{8:8}$	a	Ubiquinone	26.33	$C_{49}H_{75}O_4$	H^+	727.566	1.21
$UQ_{8:8}$	b	Ubiquinone	26.82	$C_{49}H_{75}O_4$	H^+	727.566	1.27
$UQ_{8:8}$	c	Ubiquinone	28.74	$C_{49}H_{75}O_4$	H^+	727.566	-0.88
$MQ_{8:7}$		Ubiquinone	29.91	$C_{50}H_{80}O_4N$	NH_4^+	758.608	-1.10
$UQ_{9:9}$	b	Ubiquinone	33.50	$C_{54}H_{83}O_4$	H^+	795.629	-0.02
$UQ_{10:10}$	a	Ubiquinone	35.87	$C_{59}H_{94}O_4N$	NH_4^+	880.718	-0.39

$UQ_{10:10}$	b	Ubiquinone	38.08	$C_{59}H_{94}O_4N$	NH_4^+	880.718	-0.22
$MK_{6:6}$		Menaquinone	25.33	$C_{41}H_{60}O_2N$	NH_4^+	598.461	-0.94
$MK_{7:7}$		Menaquinone	30.37	$C_{46}H_{68}O_2N$	NH_4^+	666.524	-1.10
$MK_{8:8}$		Menaquinone	35.45	$C_{51}H_{76}O_2N$	NH_4^+	734.587	-0.14

Supplementary Table 4. Quinones identified in this study for *Methylotenera mobilis* (DSM 17540) where MQ = methylene-ubiquinone and UQ = ubiquinone. Isomers are indicated as "a", "b", and "c" based on retention times for all quinones identified in the three cultures.

Quinone	Isomer	Headgroup	tr (min)	AEC	MS^2 ion	$M_{calc}(m/z)$	Δppm
$UQ_{7:7}$	a	Ubiquinone	22.35	$C_{44}H_{67}O_4$	H^+	659.503	-0.79
$UQ_{7:7}$	b	Ubiquinone	24.08	$C_{44}H_{67}O_4$	H^+	659.503	-0.97
$UQ_{8:8}$+OCH_3	a	Ubiquinone	26.47	$C_{50}H_{77}O_5$	H^+	757.577	0.11
$UQ_{8:8}$	b	Ubiquinone	26.73	$C_{49}H_{75}O_4$	H^+	727.566	0.17
$UQ_{8:8}$	c	Ubiquinone	28.73	$C_{49}H_{75}O_4$	H^+	727.566	0.39
$UQ_{9:9}$	b	Ubiquinone	33.53	$C_{54}H_{83}O_4$	H^+	795.629	-0.02
$UQ_{10:10}$	b	Ubiquinone	38.11	$C_{59}H_{94}O_4N$	NH_4^+	880.718	1.86

Supplementary Table 5. Quinones identified in this study for *Methylovulum psychrotolerans* where MQ = methylene-ubiquinone and UQ = ubiquinone. Isomers are indicated as "a", "b", and "c" based on retention times for all quinones identified in the three cultures.

Quinone	Isomer	Headgroup	tr (min)	AEC	MS^2 ion	$M_{calc}(m/z)$	Δppm
$UQ_{7:7}$	a	Ubiquinone	22.29	$C_{44}H_{67}O_4$	H^+	659.503	0.51
$UQ_{7:7}$	b	Ubiquinone	23.97	$C_{44}H_{67}O_4$	H^+	659.503	0.04
$UQ_{8:8}$	b	Ubiquinone	26.72	$C_{49}H_{75}O_4$	H^+	727.566	1.01
$UQ_{8:8}$	c	Ubiquinone	28.68	$C_{49}H_{75}O_4$	H^+	727.566	0.60
$UQ_{9:9}$	a	Ubiquinone	31.35	$C_{54}H_{83}O_4$	H^+	795.629	2.75
$UQ_{9:9}$	b	Ubiquinone	33.49	$C_{54}H_{83}O_4$	H^+	795.629	0.49
$UQ_{10:10}$	b	Ubiquinone	38.07	$C_{59}H_{94}O_4N$	NH_4^+	880.718	0.30

3.2 Identification of quinones

Quinones were identified based on diagnostic headgroup fragments and their elemental composition. Partial chromatograms are shown for quinones identified in *Methylobacter-Methylotenera* enrichment culture: $UQ_{8:8}$+OCH_3+OH (see below for identification), $UQ_{7:7}$, $UQ_{8:8}$+OCH_3 (see below for identification), $UQ_{8:8}$, $MQ_{8:7}$, $UQ_{9:9}$, and $UQ_{10:10}$ (Fig. S3).

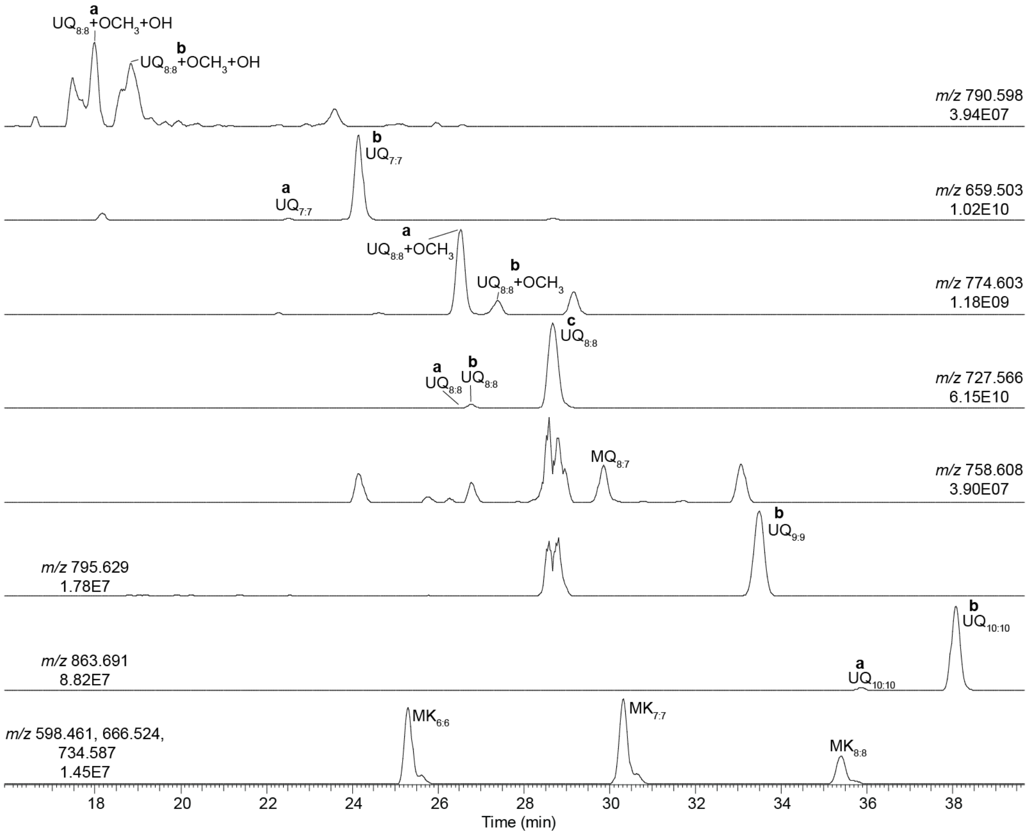

Supplementary Figure 3. Partial chromatograms of quinones identified in *Methylobacter-Methylotenera* enrichment culture with the isomers indicated by "a", "b", and "c". Note, for several quinones the isomers do not include "a" because this isomer was not identified in this enrichment culture but were found in other cultures analyzed in this study. The same isomer naming is used to ensure consistency between cultures.

A search for *m/z* 197.081 (ubiquinone head group) revealed several novel quinones in the enrichment co-culture. In the partial mass chromatogram of *m/z* 790.598 (assigned elemental composition (AEC) $C_{50}H_{80}O_6N^+$) we identified two peaks at 18.00 (Δppm -0.50) and 18.87 mins (Δppm -0.49; Fig. S4). Fragments observed in the MS^2 spectrum include *m/z* 741.545 ($C_{49}H_{73}O_5^+$, Δppm 0.22) from a loss of 32 Da from the H^+ ion, *m/z* 773.571 ($C_{50}H_{77}O_6^+$, Δppm 0.32). This is followed by a hydroxyl loss that yields the fragment *m/z* 723.535 ($C_{49}H_{71}O_4^+$, Δppm -0.74). Based on the elemental composition and the diagnostic fragment ion, *m/z* 197.081 ($C_{10}H_{13}O_4^+$, Δppm -1.19 and 0.078 for the first and second peak, respectively), we tentatively identify this as an ubiquinone with 8 isoprenoid units and 8 unsaturations with an additional methoxy group and one hydroxyl group on the side-chain. Therefore, we putatively name this compound $UQ_{8:8}$+OCH_3+OH.

Two peaks were observed in the partial mass chromatogram of *m/z* 774.603 (AEC $C_{50}H_{80}O_5N^+$) at 26.56 (Δppm -0.92) and 27.39 mins (Δppm 0.16; Fig. S5a). The dominant fragment for each peak was *m/z* 197.081 ($C_{10}H_{13}O_4^+$, Δppm -0.73 and -0.58 for the first and second peak, respectively), which indicates a ubiquinone headgroup (Fig. S5b). We also observed *m/z* 725.551 ($C_{49}H_{73}O_4^+$, Δppm -1.51) that corresponds to a loss of 32 Da from m/z 757.579 ($C_{50}H_{76}O_5^+$, Δppm -3.23), suggesting a methoxy loss. Based on the elemental composition, we tentatively assign this as having

8 isoprenoid units, with 8 unsaturations, and a methoxy group on the side-chain. Therefore, we refer to this compound as $UQ_{8:8}$+OCH_3.

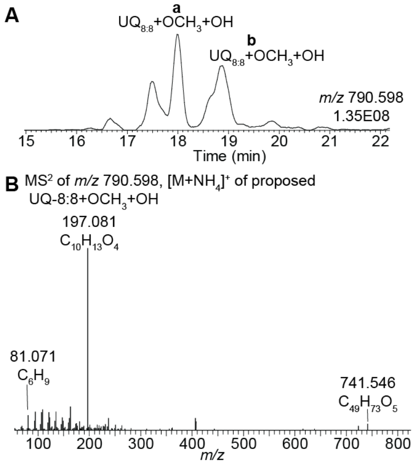

Supplementary Figure 4. (A) Partial mass chromatograms of proposed $UQ_{8:8}$+OCH_3+OH and its isomer identified in the *Methylobacter-Methylotenera* enrichments. Note, isomers are labeled as "a" and "b" as indicated in Tables S4-S6. **(B)** MS^2 spectrum of $[M+NH_4]^+$ $UQ_{8:8}$+OCH_3+OH peak.

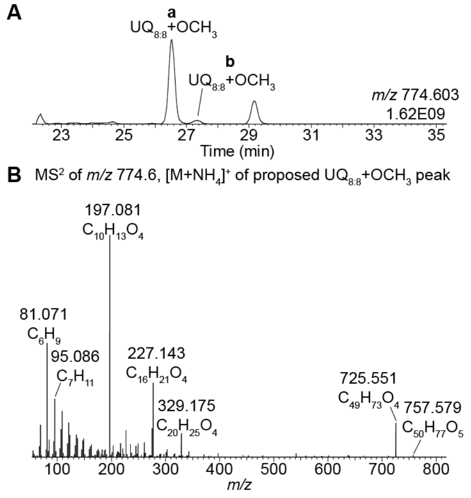

Supplementary Figure 5. (A) Partial mass chromatogram of proposed $UQ_{8:8}$+OCH_3 and its isomer identified in the *Methylobacter-Methylotenera* enrichments. Note, isomers are labeled as "a" and "b" as indicated in Tables S2-S3. **(B)** MS^2 spectrum of $[M+NH_4]^+$ $UQ_{8:8}$+OCH_3 peak.

4 Bacteriohopanepolyols (BHPs)

4.1 Summary of BHPs

Supplementary Table 6. BHPs identified in this study for the *Methylobacter-Methylotenera* enrichments. Note, the identification of unsaturation positions is based either on the MS^2 or on the difference in retention time between the unsaturated BHP and its saturated counterpart.

BHP	tr (min)	AEC	MS^2 ion	M_{calc}(*m/z*)	Δppm	References
adenosylhopane	21.77	$C_{40}H_{64}O_3N_5$	H^+	662.500	0.45	Hopmans et al. 2021
2Me-adenosylhopane$_{HG\text{-}diMe}$	25.25	$C_{43}H_{70}O_3N_5$	H^+	704.547	-0.48	Hopmans et al. 2021
aminotriol	17.52	$C_{35}H_{64}O_3N$	H^+	546.488	0.17	
Δ^6-aminotriol	15.47	$C_{35}H_{62}O_3N$	H^+	544.472	-1.12	
Δ^{11}- aminotriol	15.88	$C_{35}H_{62}O_3N$	H^+	544.472	-0.22	
$C_{14:0}$-*N*-acyl-aminotriol	33.91	$C_{49}H_{90}O_4N$	H^+	756.686	-0.77	Hopmans et al. 2021
$C_{16:0}$-*N*-acyl-aminotriol	37.41	$C_{51}H_{94}O_4N$	H^+	784.718	0.48	Hopmans et al. 2021
$C_{16:1}$-*N*-acyl-aminotriol	34.44	$C_{51}H_{92}O_4N$	H^+	782.702	0.49	Hopmans et al. 2021
aminotetrol	16.30	$C_{35}H_{64}O_4N$	H^+	562.483	-1.24	
aminopentol	14.69	$C_{35}H_{64}O_5N$	H^+	578.478	0.64	
Δ^{11}- aminopentol	13.51	$C_{35}H_{62}O_5N$	H^+	576.462	-0.57	
$C_{14:0}$-*N*-acyl-aminopentol	27.49	$C_{49}H_{90}O_6N$	H^+	788.676	-0.24	Hopmans et al. 2021
$C_{16:0}$-*N*-acyl-aminopentol	30.82	$C_{51}H_{94}O_6N$	H^+	816.708	-0.43	Hopmans et al. 2021
$C_{16:1}$-*N*-acyl-aminopentol	28.04	$C_{51}H_{92}O_6N$	H^+	814.692	0.12	Hopmans et al. 2021
$C_{16:1}$-*N*-acyl-aminopentol	28.17	$C_{51}H_{92}O_6N$	H^+	814.692	0.12	Hopmans et al. 2021
$C_{16:1}$-*N*-acyl-aminopentol	29.19	$C_{51}H_{92}O_6N$	H^+	814.692	0.12	Hopmans et al. 2021
methylcarbamate-aminotriol	20.89	$C_{37}H_{66}O_5N$	H^+	604.494	-0.22	Rush et al. 2016
methylcarbamate-aminotetrol	18.96	$C_{37}H_{66}O_6N$	H^+	620.488	-1.13	Rush et al. 2016
methylcarbamate-aminopentol	16.87	$C_{37}H_{66}O_7N$	H^+	636.483	0.57	Rush et al. 2016
ethylcarbamate-aminotriol	21.37	$C_{38}H_{68}O_5N$	H^+	618.509	-0.68	this study, Fig. S8
ethylcarbamate-aminotetrol	19.16	$C_{38}H_{68}O_6N$	H^+	634.504	-1.47	this study, Fig. S8
ethylcarbamate-aminopentol	17.05	$C_{38}H_{68}O_7N$	H^+	650.499	-1.15	this study, Fig. S8
ethenolamine-BHT	20.40	$C_{37}H_{66}O_4N$	H^+	588.499	-0.12	Hopmans et al. 2021
ethenolamine-BHhexol	16.55	$C_{37}H_{66}O_6N$	H^+	620.488	-0.76	Hopmans et al. 2021
N-formylated-aminotriol	20.28	$C_{36}H_{64}O_4N$	H^+	574.483	-1.16	Hopmans et al. 2021
N-formylated-aminopentol	16.44	$C_{36}H_{64}O_6N$	H^+	606.473	-0.24	Richter et al. 2023
aminohexol I	13.08	$C_{35}H_{64}O_6N$	H^+	594.473	-0.63	Richter et al. 2023
aminohexol II	13.94	$C_{35}H_{64}O_6N$	H^+	594.473	-0.68	Richter et al. 2023

oxazinone-aminotriol	20.82	$C_{36}H_{62}O_4N$	H^+	572.467	0.23	Elling et al. 2022; Richter et al. 2023
dioxanone-methylaminotriol I	21.52	$C_{37}H_{64}O_4N$	H^+	586.483	1.37	Richter et al. 2023
dioxanone-methylaminotriol II	22.54	$C_{37}H_{64}O_4N$	H^+	586.483	0.23	Richter et al. 2023
unknown composite	16.25	$C_{37}H_{64}O_6N$	H^+	618.473	0.61	this study, Fig. S7

Supplementary Table 7. BHPs identified in this study for *Methylovulum psychrotolerans*. Note, the identification of unsaturation positions for aminotriol, aminotetrol, and aminopentol were previously determined using EI mass spectra of hopanols formed by Rohmer degradation (Bale et al., 2019). Unknown unsaturation positions are listed as unsaturated (unsat.).

BHP	tr (min)	AEC	MS^2 ion	M_{calc}(*m/z*)	Δppm	References
aminotriol	18.16	$C_{35}H_{64}O_3N$	H^+	546.488	-0.22	
Δ^{11}- aminotriol	16.37	$C_{35}H_{62}O_3N$	H^+	544.472	-0.06	
$C_{14:0}$-*N*-acyl-aminotriol	33.70	$C_{49}H_{90}O_4N$	H^+	756.686	0.44	Hopmans et al. 2021
$C_{16:0}$-*N*-acyl-aminotriol	37.24	$C_{51}H_{94}O_4N$	H^+	784.718	0.42	Hopmans et al. 2021
$C_{16:1}$-*N*-acyl-aminotriol	33.98	$C_{51}H_{92}O_4N$	H^+	782.702	0.97	Hopmans et al. 2021
$C_{16:1}$-*N*-acyl-aminotriol	34.29	$C_{51}H_{92}O_4N$	H^+	782.702	0.54	Hopmans et al. 2021
$C_{16:1}$-*N*-acyl-aminotriol	35.05	$C_{51}H_{92}O_4N$	H^+	782.702	1.25	Hopmans et al. 2021
$C_{16:2}$-*N*-acyl-aminotriol	30.44	$C_{51}H_{90}O_4N$	H^+	780.686	0.42	Hopmans et al. 2021
aminotetrol	16.80	$C_{35}H_{64}O_4N$	H^+	562.483	0.18	
Δ^{11}-aminotetrol	15.36	$C_{35}H_{62}O_4N$	H^+	560.467	-0.04	
aminopentol	15.19	$C_{35}H_{64}O_5N$	H^+	578.478	0.38	
Δ^{11}-aminopentol	13.83	$C_{35}H_{62}O_5N$	H^+	576.462	0.03	
methylcarbamate-aminotriol	20.63	$C_{37}H_{66}O_5N$	H^+	604.494	0.22	Rush et al. 2016
unsat. methylcarbamate-aminotriol	18.33	$C_{37}H_{64}O_5N$	H^+	602.478	0.02	Rush et al. 2016
methylcarbamate-aminotetrol	18.71	$C_{37}H_{66}O_6N$	H^+	620.488	0.05	Rush et al. 2016
unsat. methylcarbamate-aminotetrol	16.78	$C_{37}H_{64}O_6N$	H^+	618.473	0.05	Rush et al. 2016
methylcarbamate-aminopentol	16.63	$C_{37}H_{66}O_7N$	H^+	636.483	0.05	Rush et al. 2016
unsat. methylcarbamate-aminopentol	15.11	$C_{37}H_{64}O_7N$	H^+	634.468	0.55	Rush et al. 2016
ethenolamine-BHT	20.42	$C_{37}H_{66}O_4N$	H^+	588.499	0.20	Hopmans et al. 2021
unsat. ethenolamine-BHT	18.11	$C_{37}H_{64}O_4N$	H^+	586.483	0.96	Richter et al. 2023
N-formylated-aminotriol	20.16	$C_{36}H_{64}O_4N$	H^+	574.483	-0.41	Hopmans et al. 2021
unsat. *N*-formylated-aminotriol	17.88	$C_{36}H_{62}O_4N$	H^+	572.467	-1.30	Richter et al. 2023
N-formylated-aminotetrol	18.26	$C_{36}H_{64}O_5N$	H^+	590.478	1.44	Hopmans et al. 2021
N-formylated-aminopentol	16.26	$C_{36}H_{64}O_6N$	H^+	606.473	0.21	Richter et al. 2023
oxazinone-aminotriol	20.57	$C_{36}H_{62}O_4N$	H^+	572.467	0.08	Elling et al. 2022; Richter et al. 2023
unknown composite	20.07	$C_{39}H_{68}O_7N$	H^+	662.500	1.44	this study, Fig. S8

4.2 Identification of novel BHPs

4.2.1 Ethylcarbamte-aminoBHPs

In the *Methylobacter-Methylotenera* enrichments grown at 2, 4, and 10 g/L NaCl we observed additional peaks in the chromatograms of *m/z* 618.509, 634.504, 650.499 while searching for methylated versions of the MC-aminoBHPs (Fig. S6a). The MS^2 spectrum of *m/z* 618.509 (AEC $C_{38}H_{68}O_5N^+$, Δppm -0.68; Fig. S6b), shows a loss of three hydroxyl moieties to yield *m/z* 600.499 ($C_{38}H_{66}O_4N^+$, Δppm 0.23), *m/z* 582.488 (AEC $C_{38}H_{64}O_3N^+$, Δppm 0.79), and *m/z* 564.477 ($C_{38}H_{62}O_2N^+$, Δppm 0.23). We also observe the fragment *m/z* 475.430 ($C_{35}H_{55}^+$, Δppm -0.42), which suggests that the additional methylation does not occur on the core structure of the compound. Instead of the expected loss of the methylcarbamate group (75 Da), we observe a loss of 89 Da. This suggests that the methylation occurs on the methylcarbamate moiety. This is supported by the presence of *m/z* 102.055 ($C_4H_8O_2N^+$, ppm -4.16) and *m/z* 132.066 ($C_5H_{10}O_3N^+$, ppm -1.82), which are equivalent to *m/z* 88 and *m/z* 118 ions in the methylcarbamate-aminotriol MS^2 spectrum but with an additional 14 Da. We, therefore, tentatively identify this compound as 35-ethylcarbamate-aminobacteriohopane-32,33,34-triol (EC-aminotriol from herein). We observe similar fragmentations in the MS^2 spectrum of *m/z* 650.499, which we putatively identify as 35-ethylcarbamate-aminobacteriohopane-30,31,32,33,34-pentol (EC-aminopentol from herein). respectively. We do not have a clear MS^2 spectrum for *m/z* 634.504, therefore we tentatively identify the peak as 35-ethylcarbamate-aminobacteriohopane-31,32,33,34-tetrol (EC-aminotetrol from herein) based on the retention time and the MS^1 spectrum.

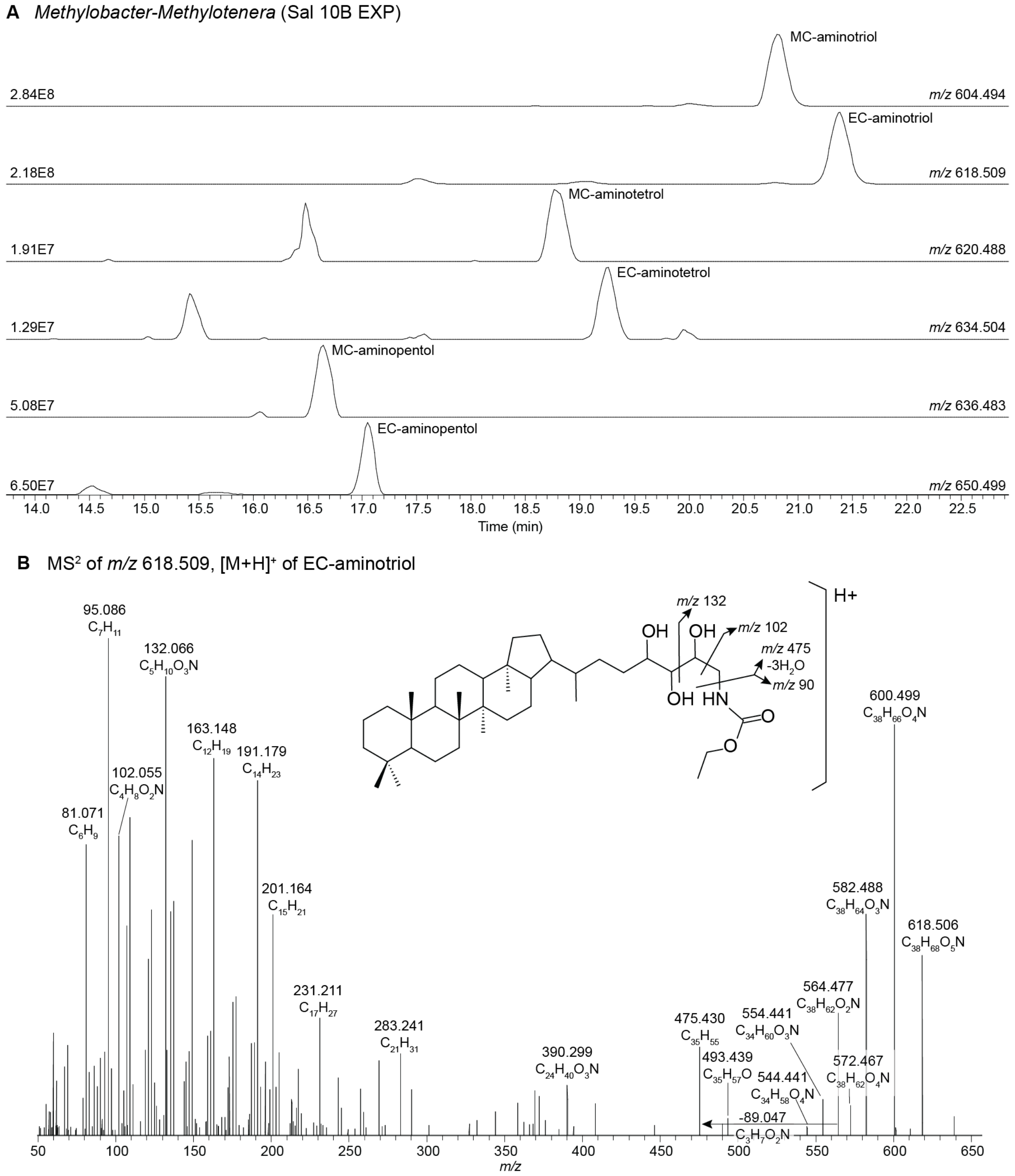

Supplementary Figure 6. (A) Partial mass chromatograms of methylcarbamate (MC) and ethylcarbamate (EC)-amino BHPs from the *Methylobacter-Methylotenera* adapted to NaCl 10 g/L conditions showing the exact mass and intensity of the highest peak in arbitrary units (AU) **(B)** MS^2 of novel EC-aminotriol and the proposed structure.

4.2.2 Unknown composite BHP in the *Methylobacter-Methylotenera* enrichments

In the partial mass chromatogram of *m/z* 618.473 we detected a peak at 16.19 mins in the *Methylobacter-Methylotenera* enrichments that were grown with 4 g/L NaCl in NMS media and no methane addition (0% CH_4). The MS^2 spectrum of *m/z* 618.473 (AEC $C_{37}H_{64}O_6N^+$, Δppm 0.61; Fig. S7) shows a loss of four hydroxyl groups yielding: *m/z* 600.462 ($C_{37}H_{62}O_5N^+$, Δppm -0.20), *m/z* 582.452 ($C_{37}H_{60}O_4N^+$, Δppm -1.04), *m/z* 564.440 ($C_{37}H_{58}O_3N^+$, Δppm 1.81), and *m/z* 546.432 ($C_{37}H_{56}O_2N^+$, Δppm -3.06). In addition, there is a loss of 28 Da (CO) from *m/z* 618.473 that yields *m/z* 590.477 ($C_{36}H_{64}O_5N^+$, Δppm 1.41) followed by the loss of three hydroxyl moieties: *m/z* 572.467 ($C_{36}H_{62}O_4N^+$, Δppm 0.13), *m/z* 554.457 ($C_{36}H_{60}O_3N^+$, Δppm -0.79), and *m/z* 536.444 ($C_{36}H_{58}O_2N^+$, Δppm 4.26). Further, we detect *m/z* 469.384 ($C_{35}H_{49}^+$, Δppm -2.82) and *m/z* 397.383 ($C_{29}H_{49}^+$, Δppm -1.24) that indicates a loss of seven functional groups from the side-chain and no modifications to the core structure, respectively.

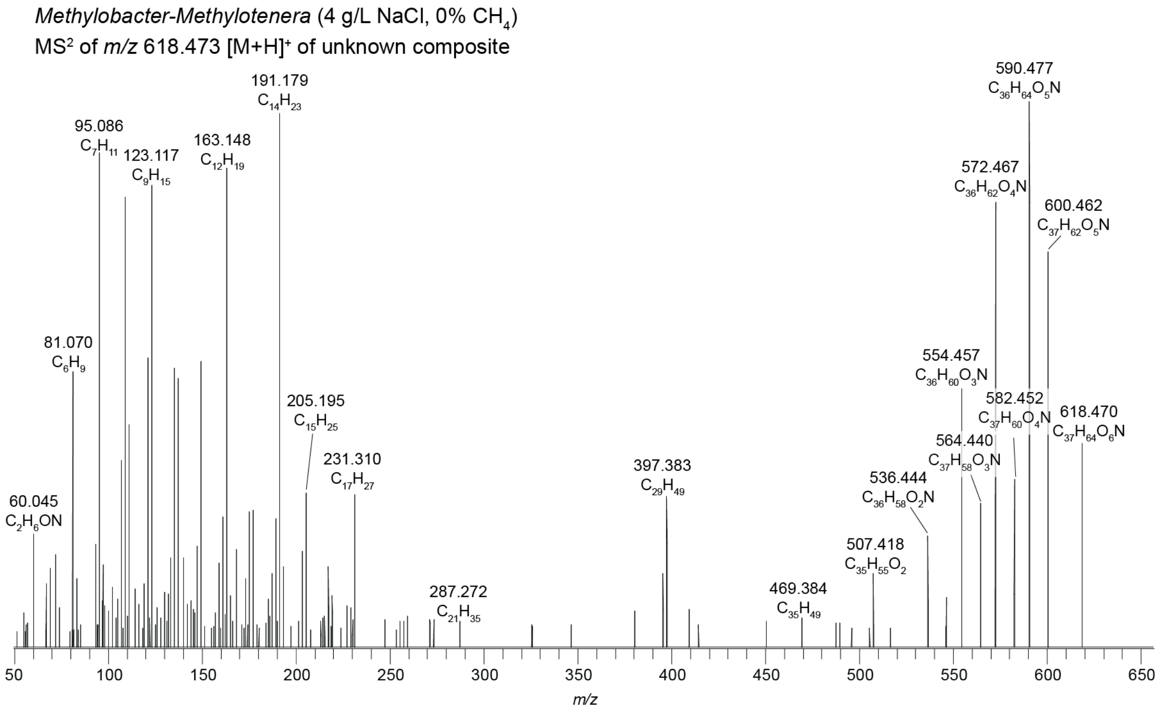

Supplementary Figure 7. MS^2 of novel unknown composite identified in *Methylobacter-Methylotenera* enrichments with NaCl additions.

4.2.3 Unknown composite BHP in *Methylovulum psychrotolerans*

In the partial mass chromatogram of adenosylhopane (*m/z* 662.501) for *Methylovulum psychrotolerans* we detected an additional peak at 20.07 min. The MS^2 spectrum of *m/z* 662.499 (AEC $C_{39}H_{68}O_7N^+$, Δppm -0.62; Fig. S8) shows a loss of three hydroxyl moieties resulting in *m/z* 644.488 ($C_{39}H_{66}O_6N^+$, Δppm 1.45), *m/z* 626.480 ($C_{39}H_{64}O_5N^+$, Δppm -3.02), and *m/z* 608.468 ($C_{39}H_{62}O_4N^+$, Δppm -1.29). A loss of 90 Da ($C_3H_6O_3$) from *m/z* 662.499 yields *m/z* 572.466 ($C_{36}H_{62}O_4N^+$, Δppm 1.56), and we observe the corresponding ion *m/z* 91.040 ($C_3H_7O_3^+$, Δppm -6.14). We also observe a*m/z* 475.430 ($C_{35}H_{55}^+$, Δppm 0.67), which confirms the loss of four functional groups from the side-chain.

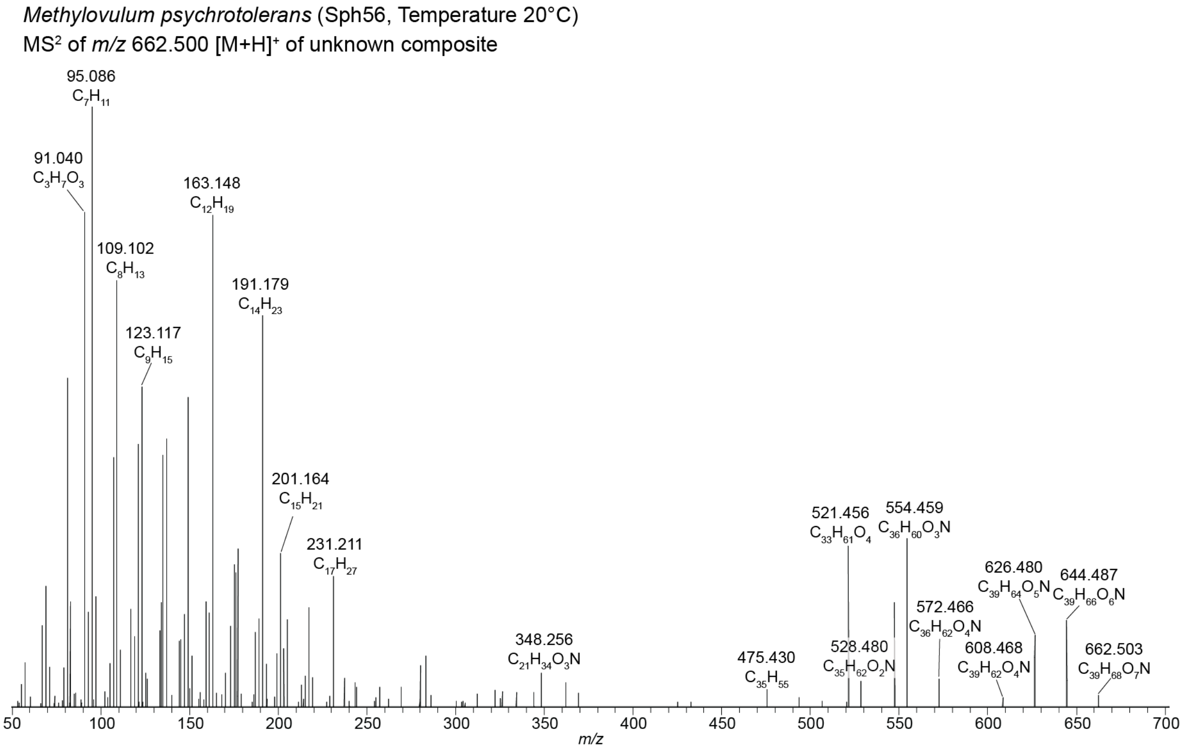

Supplementary Figure 8. MS^2 of novel unknown composite BHP identified in incubations with *Methylovulum psychrotolerans*.

5 References

Bale, N. J., Rijpstra, W. I. C., Sahonero-Canavesi, D. X., Oshkin, I. Y., Belova, S. E., Dedysh, S. N., et al. (2019). Fatty Acid and Hopanoid Adaption to Cold in the Methanotroph *Methylovulum psychrotolerans*. *Front. Microbiol.* 10. Available at: https://www.frontiersin.org/articles/10.3389/fmicb.2019.00589 (Accessed October 4, 2022).

Hopmans, E. C., Smit, N. T., Schwartz-Narbonne, R., Sinninghe Damsté, J. S., and Rush, D. (2021). Analysis of non-derivatized bacteriohopanepolyols using UHPLC-HRMS reveals great structural diversity in environmental lipid assemblages. *Org. Geochem.* 160, 104285. doi: 10.1016/j.orggeochem.2021.104285

Richter, N., Hopmans, E. C., Mitrović, D., Raposeiro, P. M., Gonçalves, V., Costa, A. C., et al. (2023). Distributions of bacteriohopanepolyols in lakes and coastal lagoons of the Azores Archipelago. *Biogeosciences* 20, 2065–2098. doi: 10.5194/bg-20-2065-2023

Rush, D., Osborne, K. A., Birgel, D., Kappler, A., Hirayama, H., Peckmann, J., et al. (2016). The Bacteriohopanepolyol Inventory of Novel Aerobic Methane Oxidising Bacteria Reveals New Biomarker Signatures of Aerobic Methanotrophy in Marine Systems. *PLOS ONE* 11, e0165635. doi: 10.1371/journal.pone.0165635
